# New Acylglucoses from *Solanum atriplicifolium* as Multidrug Resistance Reversal Agents Targeting Cdr1 and Mdr1

**DOI:** 10.3390/plants15162523

**Published:** 2026-08-20

**Authors:** Florimar Gil, Erika Ripani, Mariana Belén Joray, María Paula del Valle, Richard D. Cannon, Constantinos Athanassopoulos, D. Mariano A. Vera, María Cecilia Carpinella

**Affiliations:** 1 Fine Chemical and Natural Products Laboratory, CIDIE CONICET-UCC, Universidad Católica de Córdoba, Córdoba X5016DHK, Argentina; 2 Department of Chemistry and Biochemistry, College of Exact and Natural Sciences, National University of Mar del Plata—QUIAMM—INBIOTEC CONICET, Mar del Plata B7602AYL, Argentina; 3 Sir John Walsh Research Institute, School of Dentistry, University of Otago, P.O. Box 647, Dunedin 9054, New Zealand; richard.cannon@otago.ac.nz; 4 Laboratory of Synthetic Organic Chemistry, Department of Chemistry, University of Patras, GR-26504 Patras, Greece; 5 Institute of Chemistry and Biochemistry of Mar del Plata (IQUIBIM), Universidad Nacional de Mar del Plata, Mar del Plata 7600, Argentina

**Keywords:** efflux pumps, antifungal resistance, fluconazole, Mdr1, Cdr1, *Candida*, pump inhibitors

## Abstract

The increasing prevalence of multidrug resistance (MDR) in *Candida* species poses a major obstacle to effective antifungal therapy. Since the efflux pumps Cdr1 and Mdr1 are key mediators of the MDR phenotype, particularly by conferring resistance to azoles, their inhibition has emerged as a promising strategy to restore antifungal susceptibility. Bioassay-guided fractionation of *Solanum atriplicifolium*, selected based on its ability to inhibit Mdr1 and Cdr1, led to the isolation of six previously undescribed acylhexoses, including four acylglucoses, named atriplicifolin A (**1**), atriplicifolin B (**2**), atriplicifolin C (**3**) and atriplicifolin D (**4**) and two acylinositols, named atriplicifolin E (**5**) and atriplicifolin F (**6**). Among these, compounds **3** and **4** successfully restored fluconazole sensitivity in resistant *Saccharomyces cerevisiae* strains overexpressing Mdr1 (AD/CaMDR1) and Cdr1 (AD/CaCDR1), with greater activity against the latter, with minimum effective concentrations (MECs) of 6.2 and 7.5 µM, respectively. Both compounds also significantly increased the intracellular accumulation of Nile red and rhodamine 6G in AD/CaMDR1 and AD/CaCDR1 strains, again showing a stronger effect in the Cdr1-overexpressing strain, where MEC values ranged from 3.1 to 15.5 µM. Molecular dynamics simulations supported these results by revealing that compounds **3** and **4** displayed higher binding affinity for Cdr1 than fluconazole, Nile red or rhodamine 6G. Furthermore, analysis of the persistence and energetic contribution of individual ligand–transporter interactions over time, particularly within specific regions of the transporter, provided valuable insights for the rational design of novel inhibitors. Overall, these findings identify compounds **3** and **4** as promising scaffolds for the development of Mdr1 and Cdr1 modulators aimed at overcoming fungal multidrug resistance.

## 1. Introduction

Fungal infections have emerged as a major global health concern, affecting millions of individuals worldwide [1]. Despite causing an estimated 1.7 million deaths annually, comparable to tuberculosis, and nearly three times higher than malaria, these infections remain largely neglected, with limited therapeutic options [2]. Within the phylum Ascomycota, which comprises both primary and opportunistic human pathogens, the genus *Candida* is particularly notable for its prevalence and clinical significance [3,4]. Asymptomatic carriage of *Candida* is common, with colonization rates ranging from 25% to 75% in healthy individuals [5]. However, several risk factors, particularly those associated with impairment of the host immune system, may promote the transition from commensalism to invasive disease [4,6]. Invasive candidiasis, including candidemia, is associated with organ damage or failure [7,8] and high mortality rates of 30–40% [9]. Approximately six *Candida* species have been most commonly implicated in human candidiasis [10] with *C. albicans* remaining the leading etiological agent, followed by *C. glabrata* (currently classified as *Nakaseomyces glabrata*) [7,11]. Reflecting their clinical relevance, both species were included in the first World Health Organization (WHO) fungal priority pathogens list, highlighting the urgent need for effective therapeutic strategies [12]. Azoles have long been established as first-line antifungal agents due to their broad spectrum of activity and favorable pharmacological properties [9]. However, their extensive use has driven the emergence of acquired resistance and reduced susceptibility [13] not only to fluconazole (FCZ), the first-line antifungal agent for the treatment of candidiasis, but also to other azoles, resulting in the development of multidrug resistance (MDR) [14]. Among the mechanisms underlying MDR, reduced intracellular accumulation of antifungal agents due to the overexpression of efflux pumps is particularly prominent [15,16]. These transporters belong mainly to the ATP-binding cassette (ABC) and major facilitator superfamily (MFS) families [17]. ABC transporters use ATP hydrolysis to actively export substrates, whereas MFS transporters rely on the electrochemical gradient across the plasma membrane [18]. In *C. albicans*, the ABC transporters Cdr1 and Cdr2 (subfamily ABCG) are major contributors to azole resistance, while the MFS transporter Mdr1 is strongly associated with clinically relevant resistance phenotypes [14].

Given the central role of these transporters in the MDR phenotype, considerable efforts have focused on developing strategies to inhibit their transport function. Among these, transporter inhibitors have emerged as one of the most promising approaches to restore antifungal susceptibility [19]. In this context, plant-derived compounds have attracted increasing attention as potential adjuvants capable of reversing resistance by modulating the transporter activity [20,21,22].

With the aim of identifying compounds capable of circumventing MDR through modulation of Cdr1 and Mdr1 in resistant *Candida* strains, *Solanum atriplicifolium* was selected as a promising source, based on its previously reported efflux pump inhibitory activity [23]. Bioassay-guided fractionation of its extract led to the isolation of four novel acylglucoses with inhibitory activity against both transporters, whose potency and mechanisms of action as MDR reversal agents were subsequently investigated.

## 2. Results and Discussion

### 2.1. Active Principles from Solanum Atriplicifolium

**Figure 1:**
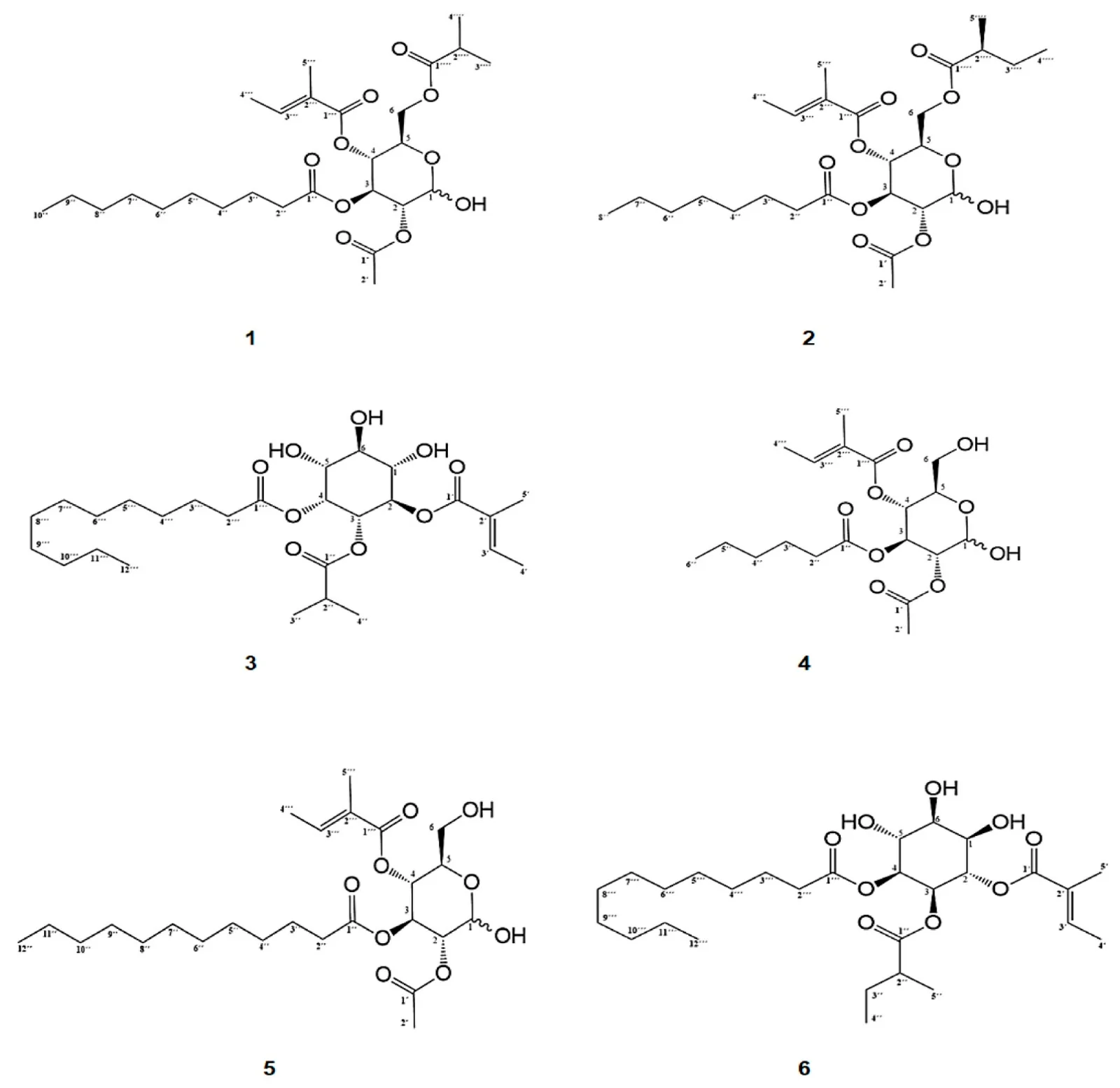
Chemical structures of atriplicifolin A (**1**), atriplicifolin B (**2**), atriplicifolin C (**3**), atriplicifolin D (**4**), atriplicifolin E (**5**) and atriplicifolin F (**6**).

Our previous work confirmed *S. atriplicifolium* as a promising source of inhibitors targeting Cdr1 and Mdr1 transporters [23]. Therefore, its ethanol extract was submitted to bioassay-guided fractionation, leading to the isolation of six new acylhexoses (Figure 1).

The chemical structures of these were elucidated through comprehensive analyses of 1D and 2D NMR spectroscopic data and supported by comparison with previously reported compounds [24,25,26,27]. The separation of these metabolites proved challenging, as previously described for structurally related acyl sugars [24,25]. Four compounds were characterized as acylglucoses and, as new compounds, were designated atriplicifolin A (**1**), atriplicifolin B (**2**), atriplicifolin C (**3**) and atriplicifolin D (**4**) (Figure 1, Table 1 and Table 2). The remaining two compounds were identified as acylinositol derivatives and named atriplicifolin E (**5**) and atriplicifolin F (**6**) (Figure 1 and Table 3). The occurrence of acylsugars with a hexose core esterified with different acyl chains, has previously been documented in the Solanaceae family although only in a few species [26,28]. To the best of our knowledge, this is the first study to describe these structures and their anti-MDR activity, thereby expanding current knowledge of the phytochemical and biological properties of this botanical family, particularly with respect to the underexplored species *S. atriplicifolium*. 

**Figure 2:**
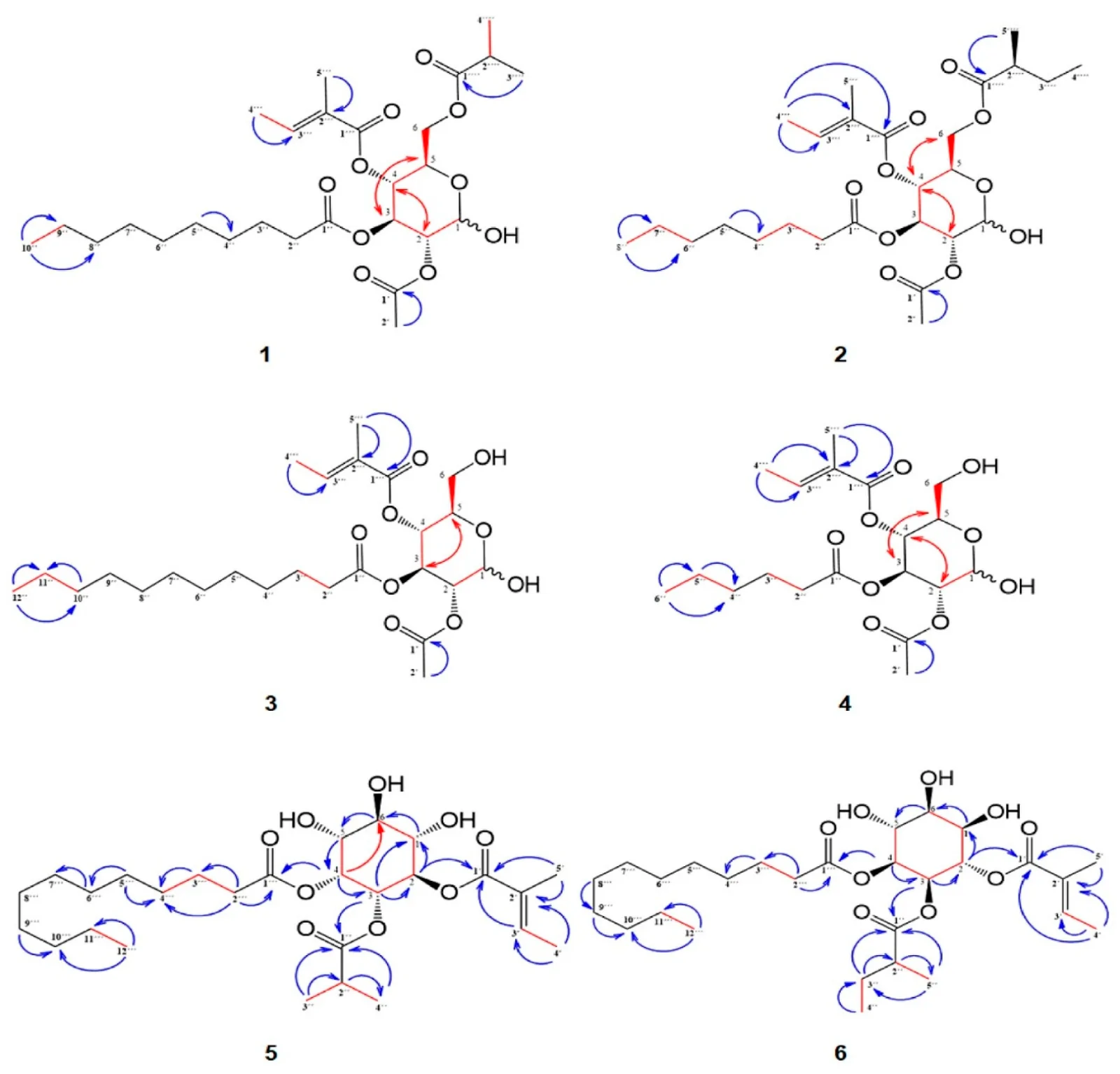
Major correlations observed in the HMBC (blue arrows) and COSY (red bonds and arrows) spectra of atriplicifolin A (**1**); atriplicifolin B (**2**), atriplicifolin C (**3**), and atriplicifolin D (**4**); atriplicifolin E (**5**) and atriplicifolin F (**6**).

Analysis of ^1^H and ^13^C NMR data (Table 1, Table 2 and Table 3), together with HSQC, HMBC, and COSY spectra (Figure 2), enabled the elucidation of the chemical structures and relative configurations of the isolated compounds revealing the presence of four tetraacylglucoses (**1**–**4**) and two triacylinositols (**5**–**6**). These two classes of metabolites were primarily distinguished by the presence of an anomeric carbon resonance at δ_C_ ~ 90–100 ppm in the tetraacylglucoses. Acylation occurred at C-2, C-3, C-4, and C-6 of the glucose core, and at C-2, C-3, and C-4 of the inositol ring (Figure 1 and Figure 2). The unbranched acyl substituents at the C-3 or C-4 positions in acylglucoses and acylinositols, respectively, consisted of alkyl chains of varying lengths, ranging from 6 to 12 carbons. This type of acyl chain is unusual among Solanaceae species and has been previously described only in a limited number of species such as *S. quitoense* and *S. nigra* [24,28].

Because compound **5** was obtained in the highest yield and afforded the most complete NMR dataset, its structure elucidation is described first. The remaining metabolites were then characterized by comparison with this compound. It was obtained as a clear oil, and its molecular formula was established as C_27_H_46_O_9_ by HR- ESIMS based on the sodium adduct ion at *m*/*z* 537.3033 [M + Na]^+^ (calculated for C_27_H_46_NaO_9_, *m*/*z* 537.3039) (Supplementary Material, Figure S1). The ^1^H and ^13^C NMR spectra of compound **5** (Table 3) showed characteristic signals corresponding to six oxygenated methine protons at δ_H_ 3.51 (t, *J* = 9.4 Hz, 1H, H-1), 3.68 (brs, 1H, H-6), 3.69 (d, *J* = 1.6 Hz, 1H, H-5) consistent with protons bearing free hydroxyl groups, as well as additional oxygenated methine protons at δ_H_ 5.00 (dd, *J* = 10.4, 2.8 Hz 1H, H-3), 5.36 (t, *J* = 10.0 Hz, 1H, H-2) and 5.50 (brs, 1H, H-4) corresponding to acylated oxygenated methines, correlated with carbons at δ_C_ 69.5 (C-5), 70.1 (C-3), 71.2 (C-4), 72.1 (C-2), 72.7 (C-1) and 73.2 (C-6). The presence of six oxygenated methine carbons (δC 69.5–73.2) together with the absence of an anomeric carbon signal supported the presence of an inositol core. The COSY spectrum showed correlations between H-1 to H-2, H-2 to H-3, H-3 to H-4 and H-4 to H-5 and H4 to H-6 as well as HMBC cross-peaks from H-1 to C-6, H-2 to C-1, H-3 to C-1 and to C-2, H-4 to C-3, H-5 to C-4 and H-6 to C-5, further confirmed the inositol framework and indicated esterification at C-2, C-3 and C-4 (Figure 2) [24]. The observed vicinal coupling constants were consistent with the expected chair conformation of the inositol ring. H-1 and H-2 appeared as triplets with large coupling constants (*J* = 9.4 and 10.0 Hz, respectively), consistent with trans-diaxial relationships involving both neighboring protons. H-3 showed a large coupling constant (*J* = 10.4 Hz) together with a smaller coupling (*J* = 2.8 Hz), indicating trans-diaxial and gauche vicinal interactions, respectively. A tigloyl moiety was identified by signals at δ_H_ 6.86 (dd, *J* = 7.2, 1.2 Hz, 1H, H-3′) and δ_C_ 128.1 (C-2′) and 137.6 (C-3′) along with two methyl groups at δ_H_ 1.79 (d, *J* = 7.2 Hz, 3H, H-4′) and 1.81 (brs, 3H, H-5′) and δ_C_ 12.9 (C-4′), 10.7 (C-5′) (Table 3). The HMBC correlations of H-3′to C-1′ at δ_C_ 167.3, H-4′ to C-2′ and to C-3′ and H-5′to C-1′ and C-2′, together with COSY correlation of H-3′to H-4′, confirmed the presence of this moiety (Figure 2). An HMBC correlation between H-2 and the carbonyl carbon C-1′ established that this substituent is attached at C-2 of the inositol core (Figure 2). The presence of an isobutyryl ester was supported by signals at δ_H_ 2.37 (t, *J* = 7.0 Hz, 1H, H-2″), 1.04 (d, *J* = 6.8 Hz, 3H, H-3″), 1.00 (d, *J* = 7.2 Hz, 3H, H-4″) together with δ_C_ signals at 175.9 (C-1″), 33.8 (C-2″), 17.4 (C-3″) and 17.8 (C-4″). HMBC correlations from H-3 to C-1″ carbonyl carbon confirmed attachment of this moiety at C-3 of the inositol scaffold. Additional HMBC correlations of H-2″ to C-4″, H-3″ to C-1″ and C2″ and H-4″ to C-1″ together with COSY correlations between H-2″to H-3″ and to H-4″, further supported the isobutyryl structure (Figure 2). Finally, an HMBC correlation between H-4 of the inositol ring (δ_H_ 5.50) and the carbonyl carbon at δ_C_ 173.8 (C-1′′′) (Figure 2) confirmed esterification at C-4 with a 12-carbon acyl chain, whose length was determined by mass spectrometry. This assignment was supported by characteristic methylene signals at δ_H_ 1.30–2.44, a terminal methyl group at δ_H_ 0.90 (t, *J* = 6.6 Hz, 3H, H-12″) and the carbonyl group at δ_C_ 173.8 (C-1″) (Table 3 and Figure 2).

LC/ESIMS fragmentation pattern also confirmed that compound **5** consists of an inositol core esterified with three short- or medium-chain acyl groups (Supplementary Material Figure S1). Thus, compound **5** was assigned as (1*R*,2*R*,3*R*,4*S*,5*R*,6*R*)-2,3,4-trihydroxy-6-(isobutyryloxy)-5-(((*E*)-2-methylbut-2-enoyl)oxy)cyclohexyl dodecanoate.

Compound **6** was isolated as a clear oil. The HR-ESIMS spectrum exhibited a sodiated molecular ion at *m/z* 551.3175 [M + Na]^+^ (calculated for C_28_H_48_NaO_9_, *m*/*z* 551.3195) corresponding to the molecular formula C_28_H_48_O_9_ (Supplementary Material Figure S2). The downfield chemical shifts in H-2 at δ_H_ 5.36, H-3 at δ_H_ 5.03, and H-4 at δ_H_ 5.52 suggested that compound **6** was a 2,3,4-trisubstituted inositol, closely resembling compound **5** (Table 3). The main difference between the two compounds was the presence of a 2-methylbutyryl substituent instead of an isobutyryl group, as indicated by the observed methylene and methyl resonances. The HMBC correlation of H-2″ (δ_H_ 2.20) to C-5″ (δ_C_ 15.6), H-3″ and H-5″ (δ_H_ 1.36 and 0.99) to C-1″ (δ_C_ 175.9) H-3″ to C-2″ (δ_C_ 41.1), H-4″ (δ_H_ 0.84) to C-3″ (δ_C_ 26.0), H-5″ (δ_H_ 0.99) to C-1″(δ_C_ 175.9) and H-5″to C-3″(δ_C_ 26.0) together with the COSY correlations between H-2″ to H-5″ and H-3″ to H-4″ (Figure 2) supported the presence of this moiety. The position of this substituent on the inositol ring was established by the HMBC correlation between H-3 and the carbonyl C-1″ (Figure 2). The observed vicinal coupling constants were consistent with the chair conformation of the inositol ring and supported the same relative configuration assigned for compound **5** (Table 3). The NMR and HR-ESIMS data of compound **6** identified this compound as ((1*R*,2*R*,3*R*,4*S*,5*R*,6*S*)-2,3,4-trihydroxy-5-(((*E*)-2-methylbut-2-enoyl)oxy)-6-((2-methylbutanoyl)oxy)cyclohexyl dodecanoate).

Compound **1** was isolated as a clear oil, and its molecular formula was determined as C_27_H_44_O_10_ based on the HR-ESIMS peak at *m/z* 551.2837 [M + Na]^+^ (calculated for C_27_H_44_NaO_10_, *m*/*z* 551.2831) (Supplementary Material, Figure S3). The ^1^H-NMR spectrum of compound **1** showed, four oxygenated methine protons appearing at δ_H_ 5.71 (t, *J* = 6.5 Hz, 1H, H-3*α*), 5.36 (dd, *J* = 10.8, 6.6 Hz, 1H, H-4*α*), 4.80 (1H, H-2*α*) and 3.31 (m, 1H, H-6*α*) for the *α*-anomer, and δ_H_ 5.46 (t, *J* = 10.4 Hz, 1H, H-3*β*), 5.00 (t, *J* = 7.4 Hz, 1H, H-4*β*), 4.85 (m, 1H, H-2*β*) and 3.59 (m, 1H, H-6*β*) for the *β*-anomer (Table 1). Characteristic α- and β-anomeric proton signals were observed with resonance peaks at δ_H_ 5.38 (br, 1H, H-1) and at δ_C_ 102.6 for C-1*α* and 4.43 (d, *J* = 5.2 Hz, 1H, H-1) and 100.4 for C-1*β* (Table 1). COSY correlations were observed between H-1 to H-2, H-2 to H-3 and to H-4, H-3 to H-4 and to H-5, H-4 to H-5 and H-5 to H-6 (Figure 2). The coupling constants of the four oxygenated methine protons were consistent with the expected chair conformation of the glucopyranose ring, showing two axial–equatorial and two trans-diaxial vicinal couplings. In the ^13^C spectrum, four oxygenated methine carbons corresponding to δ_C_ 63.2 (C-6*α*), 70.8 (C-3*α*), 71.3 (C-2*α*) and 71.6 (C-4*α*) for the *α*-anomer and δ_C_ 63.1 (C-6*β*), 70.6 (C-3*β*), 70.7 (C-4*β*) and 71.8 (C-2*β*) for the *β*-anomer were observed. These characteristic chemical shifts together with the presence of anomeric signals, confirmed the presence of a glucopyranose backbone. The presence of the acetyl group at the C-2 position was established based on previous literature [24] and was confirmed by observing a signal at δ_H_ 2.11 (s, 3H, H-2′) with its corresponding carbon at δ_C_ = 19.3 and a carbonyl group at δ_C_ = 170.9 (C-1′) with an HMBC correlation between H-2′ and C-1′ (Figure 2). A medium-length 10-carbon acyl chain was determined based on the characteristic signals (Table 1). The presence of a tigloyl and an isobutyryl group as substituents at C-4 and C-6, respectively, were also observed (Table 1). The combined information from NMR and LC/ESIMS fragmentation pattern led us to conclude that this compound corresponds to (3*R*,4*S*,5*R*,6*R*)-3-acetoxy-2-hydroxy-6-((isobutyryloxy)methyl)-5-(((*E*)-2-methylbut-2-enoyl)oxy)tetrahydro-2*H*-pyran-4-yl decanoate.

Compound **2** was obtained as a clear oil; its molecular formula, C_26_H_42_O_10_, was determined by HR-ESIMS with the H^+^ ion at *m*/*z* 515.2838 [M + H]^+^ (calculated for C_26_H_43_O_10_, *m/z* 515.2856) (Supplementary Material, Figure S4). This compound has a close structural resemblance to compound **1** differing only in the length of the acyl chain at C-4 (C8 instead of C10) and in the replacement of the isobutyryl moiety at C-6 by a 2-methylbutanoyl group (Table 1). This compound corresponds to (3*R*,4*S*,5*R*,6*R*)-3-acetoxy-2-hydroxy-5-(((*E*)-2-methylbut-2-enoyl)oxy)-6-(((2-methylbutanoyl)oxy)methyl)tetrahydro-2*H*-pyran-4-yl octanoate.

The absolute configuration of the stereogenic center at position 2′′′′ of the 2-methylbutanoyl moiety was not determined experimentally. However, this acyl group is biosynthetically derived from L-isoleucine, whose stereochemistry is retained during its conversion to 2-methylbutanoyl-CoA (BioCyc Database Collection, 2026). Because acylsugar acyltransferases (ASATs) transfer the intact acyl group from the corresponding acyl-CoA donor to the sugar core, the α-methyl stereocenter of the 2-methylbutanoyl substituent was assigned the (*S*)-configuration. This assignment is consistent with previous reports of structurally related acylglucoses isolated from S. *pennellii* [29].

Compound **3** was obtained as a clear oil; its molecular formula, C_25_H_42_O_9_, was determined by HR-ESIMS, which showed one [M + H]^+^ ion at *m*/*z* 487.2905 (calculated for C_25_H_43_O_9_, *m*/*z* 487.2914) (Supplementary Material, Figure S5) while compound **4** was obtained as a clear oil; its molecular formula, C_19_H_30_O_9_, was determined by HR-ESIMS with the sodiated molecular ion at *m*/*z* 425.1744 [M + Na]^+^ (calculated for C_19_H_30_NaO_9_, *m*/*z* 425.1787) (Supplementary Material, Figure S6). Both compounds shared the same acylglucose scaffold, including a hydroxyl group at C-6 and differed only in the length of the acyl chain esterifying the sugar core, corresponding to a C12 chain in compound **3** and a C6 chain in compound **4** (Table 2 and Figure 1). These metabolites were therefore characterized as (3*R*,4*S*,5*R*,6*R*)-3-acetoxy-2-hydroxy-6-(hydroxymethyl)-5-(((*E*)-2-methylbut-2-enoyl)oxy) tetrahydro-2*H*-pyran-4-yl dodecanoate and (3*R*,4*S*,5*R*,6*R*)-3-acetoxy-2-hydroxy-6-(hydroxymethyl)-5-(((*E*)-2-methylbut-2-enoyl)oxy) tetrahydro-2*H*-pyran-4-yl hexanoate, respectively.

Atriplicifolin A (α-anomer, **1**): 1H NMR (methanol-d4): δ 5.71 (t, *J* =6.5 Hz, 1H, H-3), 5.38 (br s, 1H, H-1), 5.36 (dd, *J* = 10.8, 6.6 Hz, 1H,H-4), 4.80 (m, 1H, H-2), 3.87 (m, 1H, H-5), 3.31 (m, 1H, H-6), 2.41(m, 1H, H-2′′′′), 2.27 (m, 2H, H-2′′), 2.11 (s, 3H, H-2′), 1.80 (s, 3H, H-5′′′), 1.79 (d, *J* = 4.8 Hz, 3H, H-4′′′), 1.67 (m, 2H, H-3′′), 1.29 (m,12H, H-4′′, H-5′′, H-6′′, H-7′′, H-8′′), 1.32 (m, 2H, H-9′′), 1.03 (d, *J* =4.0 Hz, 3H, H-4′′′′), 0.97 (d, *J* = 4.8 Hz, 3H, H-3′′′′), 0.90 (t, *J* = 4.6Hz, 3H, H-10′′); 13C NMR (methanol-d4): δ 176.1 (C-1′′′′), 170.9 (C-1′), 167.1 (C-1′′′), 138.6 (C-3′′′), 128.1 (C-2′′′), 102.6 (C-1), 71.6 (C-4), 71.3 (C-2), 70.8 (C-3), 68.7 (C-5), 63.2 (C-6), 33.5 (C-2′′′′), 33.3(C-2′′), 29.0 (C-4′′), 29.0 (C-5′′), 29.0 (C-6′′), 29.0 (C-7′′), 29.0 (C-8′′), 24.7 (C-3′′), 22.0 (C-9′′), 19.3 (C-2′), 17.3 (C-3′′′′), 17.1 (C-4′′′′),12.8 (C-4′′′), 12.7 (C-10′′), 10.4 (C-5′′′).

Atriplicifolin A (β-anomer, **1**): 1H NMR (methanol-d4): δ 5.46 (t, *J* =10.4 Hz, 1H, H-3), 5.00 (t, *J* = 7.4 Hz, 1H, H-4), 4.85 (m, 1H, H-2),4.43 (d, *J* = 5.2 Hz, 1H, H-1), 3.67 (m, 1H, H-5), 3.59 (m, 1H, H-6),2.41 (m, 1H, H-2′′′′), 2.27 (m, 2H, H-2′′), 2.11 (s, 3H, H-2′), 1.80 (s,3H, H-5′′′), 1.79 (d, *J* = 4.8 Hz, 3H, H-4′′′), 1.67 (m, 2H, H-3′′), 1.29 (m, 12H, H-4′′, H-5′′, H-6′′, H-7′′, H-8′′), 1.32 (m, 2H, H-9′′), 1.03 (d, *J* = 4.0 Hz, 3H, H-4′′′′), 0.97 (d, *J* = 4.8 Hz, 3H, H-3′′′′), 0.90 (t, *J* =4.6 Hz, 3H, H-10′′); 13C NMR (methanol-d4): δ 176.1 (C-1′′′′), 170.9 (C-1′), 167.1 (C-1′′′), 138.6 (C-3′′′), 128.1 (C-2′′′), 100.4 (C-1), 72.5 (C-5), 71.8 (C-2), 70.7 (C-4), 70.6 (C-3), 63.1 (C-6), 33.5 (C-2′′′′),33.3 (C-2′′), 29.0 (C-4′′), 29.0 (C-5′′), 29.0 (C-6′′), 29.0 (C-7′′), 29.0 (C-8′′), 24.7 (C-3′′), 22.0 (C-9′′), 19.3 (C-2′), 17.3 (C-3′′′′), 17.1 (C-4′′′′), 12.8 (C-4′′′), 12.7 (C-10′′), 10.4 (C-5′′′). Atriplicifolin B (α-anomer, **2**): 1H NMR (methanol-d4): δ 5.53 (m, 1H,H-3), 5.45 (t, *J* = 6.8 Hz, 1H, H-4), 4.88 (m, 1H, H-2), 4.61 (d, *J* = 4.5Hz, 1H, H-1), 3.87 (d, *J* = 2.0 Hz, 1H, H-5), 3.58 (br s, 1H, H-6), 2.37 (m, 1H, H-2′′′′), 2.33 (m, 2H, H-2′′), 2.02 (s, 3H, H-2′), 1.80 (s, 3H, H-5′′′), 1.79 (d, *J* = 3.6 Hz, 3H, H-4′′′), 1.61 (m, 2H, H-3′′), 1.48 (m, 2H,H-3′′′′), 1.29 (m, 4H, H-4′′, H-5′′), 1.28 (m, 2H, H-6′′), 1.32 (m, 2H,H-7′′), 1.00 (d, *J* = 4.4 Hz, 3H, H-5′′′′), 0.94 (t, *J* = 2.4 Hz, 3H, H-4′′′′),0.90 (t, *J* = 4.6 Hz, 3H, H-8′′); 13C NMR (methanol-d4): δ 175.9 (C-1′′′′), 170.9 (C-1′), 167.1 (C-1′′′), 138.6 (C-3′′′), 128.1 (C-2′′′), 100.3 (C-1), 71.9 (C-2), 70.8 (C-4), 70.1 (C-3), 67.8 (C-5), 63.1 (C-6), 40.9 (C-2′′′′), 33.5 (C-2′′), 31.3 (C-6′′), 29.1 (C-4′′), 29.1 (C-5′′), 26.2 (C-3′′′′),24.4 (C-3′′), 22.1 (C-7′′), 19.5 (C-2′), 15.3 (C-5′′′′), 12.8 (C-4′′′), 12.8 (C-8′′), 10.5 (C-5′′′), 10.3 (C-4′′′′).

Atriplicifolin B (β-anomer, **2**): 1H NMR (methanol-d4): δ 5.70 (m, 1H,H-3), 5.32 (t, *J* = 6.6 Hz, 1H, H-4), 4.98 (t, *J* = 1.8 Hz, 1H, H-2), 4.43 (dd, *J* = 12.8, 5.0 Hz, 1H, H-1), 3.89 (d, *J* = 1.6 Hz, 1H, H-6), 3.60 (m,1H, H-5), 2.37 (m, 1H, H-2′′′′), 2.33 (m, 2H, H-2′′), 2.11 (s, 3H, H-2′),1.80 (s, 3H, H-5′′′), 1.79 (d, *J* = 3.6 Hz, 3H, H-4′′′), 1.61 (m, 2H, H-3′′), 1.48 (m, 2H, H-3′′′′), 1.29 (m, 4H, H-4′′, H-5′′), 1.28 (m, 2H, H-6′′),1.32 (m, 2H, H-7′′), 1.00 (d, *J* = 4.4 Hz, 3H, H-5′′′′), 0.94 (t, *J* = 2.4Hz, 3H, H-4′′′′), 0.90 (t, *J* = 4.6 Hz, 3H, H-8′′); 13C NMR (methanol-d4): δ 175.9 (C-1′′′′), 170.9 (C-1′), 167.1 (C-1′′′), 138.6 (C-3′′′), 128.1 (C-2′′′), 101.3 (C-1), 73.5 (C-4), 71.4 (C-2), 71.2 (C-3), 69.5 (C-5),63.1 (C-6), 40.9 (C-2′′′′), 33.5 (C-2′′), 31.3 (C-6′′), 29.1 (C-4′′), 29.1 (C-5′′), 26.2 (C-3′′′′), 24.4 (C-3′′), 22.1 (C-7′′), 19.4 (C-2′), 15.3 (C-5′′′′), 12.8 (C-4′′′), 12.8 (C-8′′), 10.5 (C-5′′′), 10.3 (C-4′′′′).

Atriplicifolin C (α-anomer, **3**): 1H NMR (methanol-d4): δ 6.80 (dd, *J*= 6.8, 1.2 Hz, 1H, H-3′′′), 5.29 (m, 1H, H-3), 5.00 (dd, *J* = 2.8, 1.8 Hz,1H, H-4), 4.88 (d, *J* = 18.4 Hz, 1H, H-2), 4.60 (d, *J* = 2.8 Hz, 1H, H-1),3.97 (m, 1H, H-5), 3.57 (m, 2H, H-6), 2.32 (m, 2H, H-2′′), 2.06 (s, 3H,H-2′), 1.83 (s, 3H, H-5′′′), 1.81 (d, *J* = 5.2 Hz, 3H, H-4′′′), 1.61 (m,2H, H-3′′), 1.30 (m, 2H, H-4′′), 1.29 (m, 10H, H-5′′, H-6′′, H-7′′, H-8′′,H-9′′), 1.28 (m, 2H, H-10′′), 1.30 (m, 2H, H-11′′), 0.90 (t, *J* = 4.6 Hz,3H, H-12′′); 13C NMR (methanol-d4): δ 173.1 (C-1′′), 170.9 (C-1′),167.2 (C-1′′′), 136.9 (C-3′′′), 128.3 (C-2′′′), 99.8 (C-1), 73.9 (C-3),72.5 (C-5), 72.3 (C-2), 72.1 (C-4), 61.8 (C-6), 33.8 (C-2′′), 31.6 (C-10′′), 29.1 (C-4′′), 29.1 (C-5′′), 29.1 (C-6′′), 29.1 (C-7′′), 29.1 (C-8′′), 29.1 (C-9′′), 24.3 (C-3′′), 22.1 (C-11′′), 19.7 (C-2′), 12.9 (C-4′′′), 12.8 (C-12′′), 10.8 (C-5′′′).

Atriplicifolin C (β-anomer, **3**): 1H NMR (methanol-d4): δ 6.80 (dd, *J*= 6.8, 1.2 Hz, 1H, H-3′′′), 5.22 (t, *J* = 6.4 Hz, 1H, H-3), 5.02 (m, 1H, H-4), 4.96 (m, 1H, H-2), 4.54 (d, *J* = 4.4 Hz, 1H, H-1), 3.94 (m, 2H, H-6),3.51 (m, 1H, H-5), 2.32 (m, 2H, H-2′′), 2.11 (s, 3H, H-2′), 1.83 (s, 3H,H-5′′′), 1.81 (d, *J* = 5.2 Hz, 3H, H-4′′′), 1.61 (m, 2H, H-3′′), 1.30 (m,2H, H-4′′), 1.29 (m, 10H, H-5′′, H-6′′, H-7′′, H-8′′, H-9′′), 1.28 (m, 2H,H-10′′), 1.30 (m, 2H, H-11′′), 0.90 (t, *J* = 4.6 Hz, 3H, H-12′′); 13CNMR (methanol-d4): δ 173.1 (C-1′′), 170.9 (C-1′), 167.2 (C-1′′′),136.9 (C-3′′′), 128.3 (C-2′′′), 100.5 (C-1), 73.1 (C-3), 70.6 (C-4), 70.2 (C-5), 69.2 (C-2), 61.8 (C-6), 33.8 (C-2′′), 31.3 (C-10′′), 29.1 (C-4′′),29.1 (C-5′′), 29.1 (C-6′′), 29.1 (C-7′′), 29.1 (C-8′′), 29.1 (C-9′′), 24.3 (C-3′′), 22.1 (C-11′′), 19.5 (C-2′), 12.9 (C-4′′′), 12.8 (C-12′′), 10.8 (C-5′′′).

Atriplicifolin D (α-anomer, **4**): 1H NMR (methanol-d4): δ 6.80 (m,1H, H-3′′′), 5.24 (dd, *J* = 11.2, 6.4 Hz, 1H, H-3), 5.00 (m, 1H, H-4),4.88 (m, 1H, H-2), 4.61 (d, *J* = 2.8 Hz, 1H, H-1), 3.96 (m, 1H, H-5),3.57 (m, 2H, H-6), 2.33 (m, 2H, H-2′′), 2.01 (s, 3H, H-2′), 1.85 (s, 3H,H-5′′′), 1.81 (d, *J* = 5.6 Hz, 3H, H-4′′′), 1.60 (m, 2H, H-3′′), 1.26 (m,2H, H-4′′), 1.30 (m, 2H, H-5′′), 0.90 (t, *J* = 4.6 Hz, 3H, H-6′′); 13CNMR (methanol-d4): δ 173.1 (C-1′′), 170.9 (C-1′), 167.0 (C-1′′′),137.0 (C-3′′′), 128.3 (C-2′′′), 99.5 (C-1), 73.3 (C-3), 71.8 (C-4), 71.7 (C-2), 71.5 (C-5), 62.9 (C-6), 34.1 (C-2′′), 31.3 (C-4′′), 24.8 (C-3′′),21.9 (C-5′′), 19.8 (C-2′), 13.0 (C-6′′), 12.2 (C-4′′′).

Atriplicifolin D (β-anomer, **4**): 1H NMR (methanol-d4): δ 6.80 (m, 1H,H-3′′′), 5.06 (m, 1H, H-4), 4.66 (t, *J* = 4.2 Hz, 1H, H-2), 4.55 (d, *J* =4.4 Hz, 1H, H-1), 3.91 (m, 2H, H-6), 3.58 (m, 1H, H-5), 2.33 (m, 2H,H-2′′), 2.11 (s, 3H, H-2′), 1.85 (s, 3H, H-5′′′), 1.81 (d, *J* = 5.6 Hz, 3H,H-4′′′), 1.60 (m, 2H, H-3′′), 1.26 (m, 2H, H-4′′), 1.30 (m, 2H, H-5′′),0.90 (t, *J* = 4.6 Hz, 3H, H-6′′); 13C NMR (methanol-d4): δ 173.1 (C-1′′), 170.9 (C-1′), 167.0 (C-1′′′), 137.0 (C-3′′′), 128.3 (C-2′′′), 100.2 (C-1), 73.9 (C-2), 70.3 (C-4), 69.8 (C-5), 62.9 (C-6), 34.1 (C-2′′), 31.3 (C-4′′), 24.8 (C-3′′), 21.9 (C-5′′), 19.7 (C-2′), 13.0 (C-6′′), 12.2 (C-4′′′).

Atriplicifolin E (**5**): 1H NMR (methanol-d4): δ 6.86 (dd, *J* = 7.2, 1.2Hz, 1H, H-3′), 5.50 (br s, 1H, H-4), 5.36 (t, *J* = 10.0 Hz, 1H, H-2), 5.00 (dd, *J* = 10.4, 2.8 Hz, 1H, H-3), 3.69 (d, *J* = 1.6 Hz, 1H, H-5), 3.68 (brs, 1H, H-6), 3.51 (t, *J* = 9.4 Hz, 1H, H-1), 2.44 (m, 2H, H-2′′′), 2.37 (t,*J* = 7.0 Hz, 1H, H-2′′), 1.81 (br s, 3H, H-5′), 1.79 (d, *J* = 7.2 Hz, 3H,H-4′), 1.68 (m, 2H, H-3′′′), 1.41 (m, 2H, H-6′′′), 1.34 (m, 2H, H-9′′′),1.30 (m, 12H, H-4′′′, H-7′′′, H-8′′′, H-10′′′, H-11′′′), 1.31 (m, 2H, H-5′′′), 1.04 (d, *J* = 6.8 Hz, 3H, H-3′′), 1.00 (d, *J* = 7.2 Hz, 3H, H-4′′), 0.90 (t, *J* = 6.6 Hz, 3H, H-12′′′); 13C NMR (methanol-d4): δ 175.9 (C-1′′),173.8 (C-1′′′), 167.3 (C-1′), 137.6 (C-3′), 128.1 (C-2′), 73.2 (C-6),72.7 (C-1), 72.1 (C-2), 71.2 (C-4), 70.1 (C-3), 69.5 (C-5), 33.8 (C-2′′),33.8 (C-2′′′), 31.7 (C-10′′′), 29.4 (C-5′′′), 29.4 (C-8′′′), 29.2 (C-6′′′),29.1 (C-7′′′), 29.0 (C-9′′′), 28.8 (C-4′′′), 24.8 (C-3′′′), 22.3 (C-11′′′),17.8 (C-4′′), 17.4 (C-3′′), 13.0 (C-12′′′),12.9 (C-4′), 10.7 (C-5′).

Atriplicifolin F (**6**): 1H NMR (methanol-d4): δ 6.88 (dd, *J* = 7.2, 1.2Hz, 1H, H-3′), 5.52 (br s, 1H, H-4), 5.36 (t, *J* = 10.2 Hz, 1H, H-2), 5.03 (dd, *J* = 10.4, 2.8 Hz, 1H, H-3), 3.68 (d, *J* = 5.6 Hz, 1H, H-5), 3.68 (brs, 1H, H-6), 3.51 (dd, *J* = 9.6, 4.6 Hz, 1H, H-1), 2.44 (m, 2H, H-2′′′),2.20 (t, *J* = 7.0 Hz, 1H, H-2′′), 1.81 (br s, 3H, H-5′), 1.79 (d, *J* = 8.0Hz, 3H, H-4′), 1.68 (m, 2H, H-3′′′), 1.41 (m, 2H, H-6′′′), 1.36 (m, 2H,H-3′′), 1.34 (m, 2H, H-9′′′), 1.30 (m, 12H, H-4′′′, H-7′′′, H-8′′′, H-10′′′, H-11′′′), 1.31 (m, 2H, H-5′′′), 0.99 (d, *J* = 7.2 Hz, 3H, H-5′′), 0.84 (t, *J*= 7.4 Hz, 3H, H-4′′), 0.90 (t, *J* = 6.4 Hz, 3H, H-12′′′); 13C NMR (methanol-d4): δ 175.9 (C-1′′), 173.8 (C-1′′′), 167.3 (C-1′), 137.7 (C-3′), 128.1 (C-2′), 73.2 (C-6), 72.7 (C-1), 72.1 (C-2), 71.2 (C-4), 69.9 (C-3), 69.6 (C-5), 41.1 (C-2′′), 33.8 (C-2′′′), 31.7 (C-10′′′), 29.4 (C-5′′′), 29.4 (C-8′′′), 29.2 (C-6′′′), 29.1 (C-7′′′), 29.0 (C-9′′′), 28.8 (C-4′′′),26.0 (C-3′′), 24.8 (C-3′′′), 22.3 (C-11′′′), 15.6 (C-5′′), 13.0 (C-12′′′),12.9 (C-4′), 10.5 (C-4′′), 10.7 (C-5′).

### 2.2. Acylglucosides 1–4 Are Able to Restore Sensitivity to Fluconazole in Saccharomyces cerevisiae Strains Over-Expressing Mdr1 and Cdr1 Transporters

The ability of the isolated compounds to reverse fluconazole resistance was evaluated by agar-based chemosensitization assays. Compound **1** restored FCZ sensitivity in AD/CaCDR1 and AD/CaMDR1 strains with minimum effective concentration (MEC) values of 100 µM, indicating a weak activity. In contrast, compound **2** showed moderate reversal effect in both strains with MEC values of 25 µM (Table 4). Compounds **3** and compound **4** showed moderate activity against AD/CaMDR1 strain, with MEC values of 25 and 15 µM, respectively. However, they displayed markedly stronger activity against the AD/CaCDR1 strain, with MEC values of 6.2 and 7.5 µM, respectively. This differential response is consistent with a possible involvement of CDR1 in the observed phenotype. It is noteworthy that the efficacy of these two compounds against AD/CaCDR1 surpassed even that of the positive control FK506, which exhibited an MEC of 10 µM (the latter was not tested against AD/CaMDR1 due to its known exclusive selectivity for ABC transporters). In contrast, compounds **5** and **6** were inactive against both strains with MEC values ≥ 200 µM (Table 4). No antifungal activity was observed for any of the compounds when tested at 100 μM in combination with 0.5 μg/mL FCZ against the AD1-8u^−^ null mutant strain, as this FCZ concentration corresponds to one-quarter of the MIC for this strain. The differences in activity among these structurally related acylhexoses may reflect subtle variations in their substitution patterns that influence their interaction with the transporters, supporting further investigation of these compounds as potential modulators of antifungal resistance through molecular modeling studies. The results obtained showed the inositol derivatives to be completely inactive, whereas the acylglucosides displayed different levels of reversal activity. Among the latter, the primary alcohol at the C-6 position in compounds **3** and **4** favored their activity.

### 2.3. Enhancement of Nile Red Accumulation by Acylglucosides 1–4

**Figure 3:**
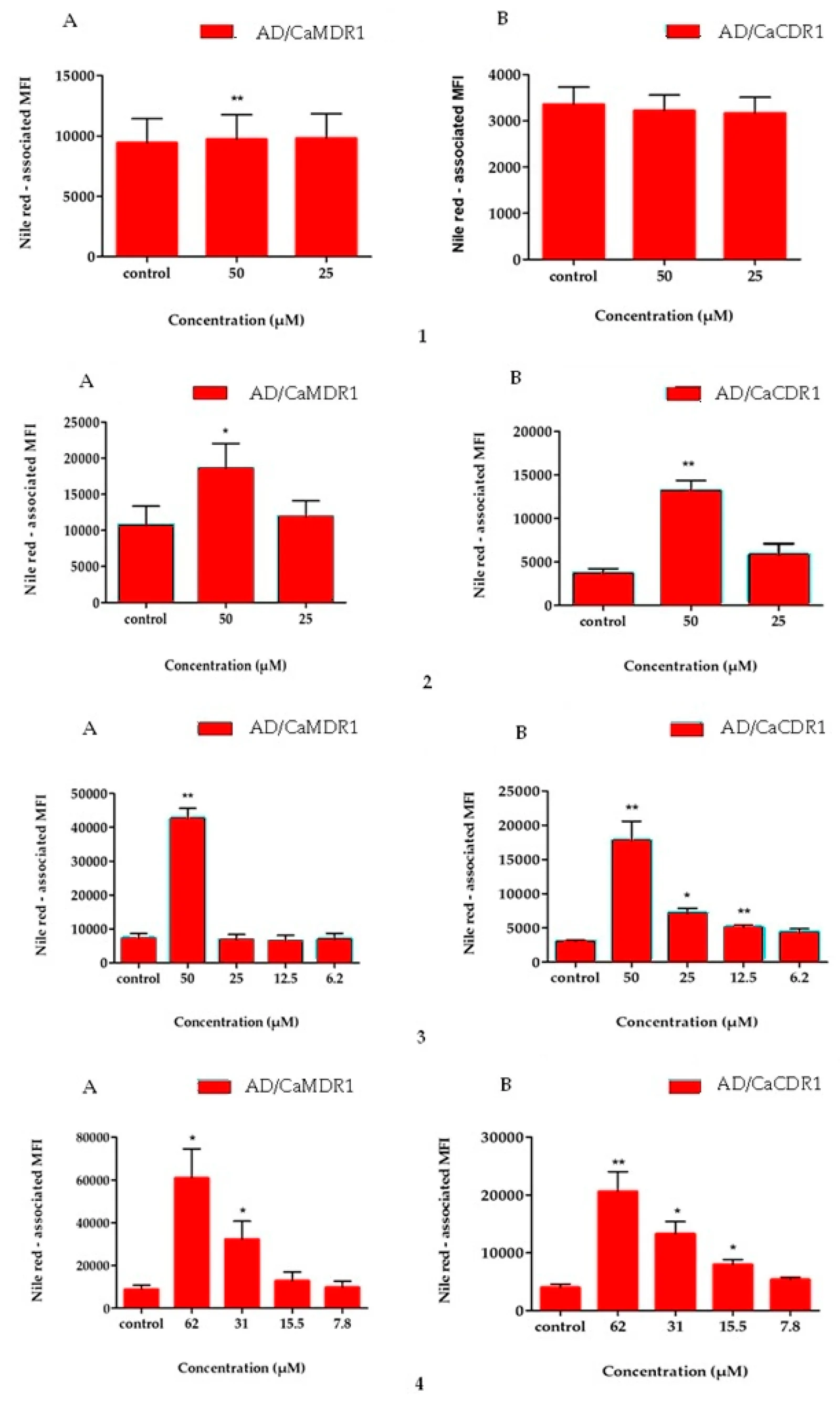
Effects of compounds **1**–**4** on the accumulation of Nile red in: (**A**) AD/CaMDR1 and (**B**) AD/CaCDR1. Significant differences compared to the negative control were determined using a one-tailed paired Student’s *t*-test (** *p* < 0.01; * *p* < 0.05).

**Figure 4:**
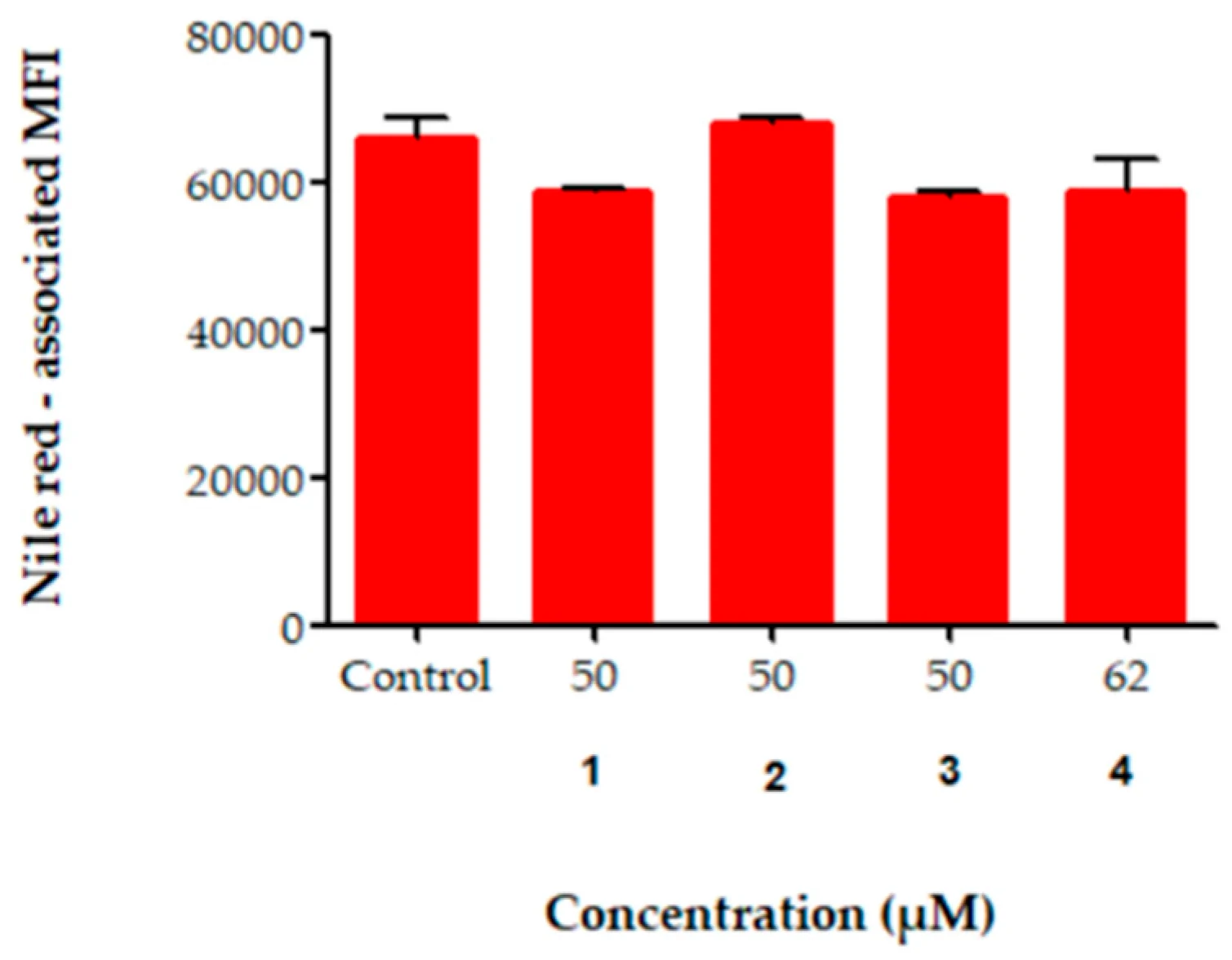
Effects of compounds **1**–**4** on Nile red accumulation in *Saccharomyces cerevisiae* AD1-8u^−^. Intracellular Nile red levels did not increase in cells treated with any of the compounds at the highest concentration tested.

To evaluate the ability of compounds **1**–**4** to increase the intracellular accumulation of the Cdr1 and Mdr1 substrate, Nile red, an accumulation assay was performed. The AD/CaMDR1 and AD/CaCDR1 strains were used, whereas the AD1-8u^−^ null mutant served as the control. Compound **1** significantly increased (*p* < 0.01) Nile red accumulation in AD/CaMDR1 cells at the maximum tested concentration of 50 µM, suggesting a selective modulation of the Mdr1 efflux pump (Table 5, Figure 3 and Figure S7). In contrast, compound **2**, significantly enhanced Nile red retention in both AD/CaMDR1 and AD/CaCDR1 cells at the same concentration (*p* < 0.05 and *p* < 0.01, respectively) (Table 5, Figure 3 and Figure S8). Compound **3** produced a moderate effect in AD/CaMDR1 cells, comparable to that observed for compounds **1** and **2**, but exhibited greater activity against AD/CaCDR1 cells, reaching an MEC value of 12.5 µM (Table 5, Figure 3 and Figure S9). Among the evaluated compounds, compound **4** displayed the strongest effect, increasing Nile red accumulation with MEC values of 31 and 15.5 µM in AD/CaMDR1 and AD/CaCDR1, respectively (Table 5, Figure 3 and Figure S10). Overall, compounds **3** and **4** showed greater enhancement of Nile red accumulation, particularly the Cdr1-overexpressing strain, consistent with the results observed in the FCZ-reversal assay. Non-significant differences (*p* > 0.05) were observed between treatments with compound **3** or compound **4** and FK506. These findings identify compounds **3** and **4** as the most potent inhibitors among the acylglucosides isolated from *S. atriplicifolium*, showing marked inhibitory activity against Cdr1. Exposure of the *S. cerevisiae* AD1-8u^−^ null mutant strain to compounds **1**–**4** at the highest tested concentrations did not result in significant increases in Nile red accumulation (Figure 4). These findings suggest that Mdr1 and Cdr1 may contribute to the observed effects, although the data do not provide direct evidence of efflux pump inhibition.

### 2.4. Inhibitory Effects on Cdr1-Mediated Outward Transport by the More Effective Compounds ***3*** and ***4*** Using the Rhodamine 6G Intracellular Accumulation Assay

**Figure 5:**
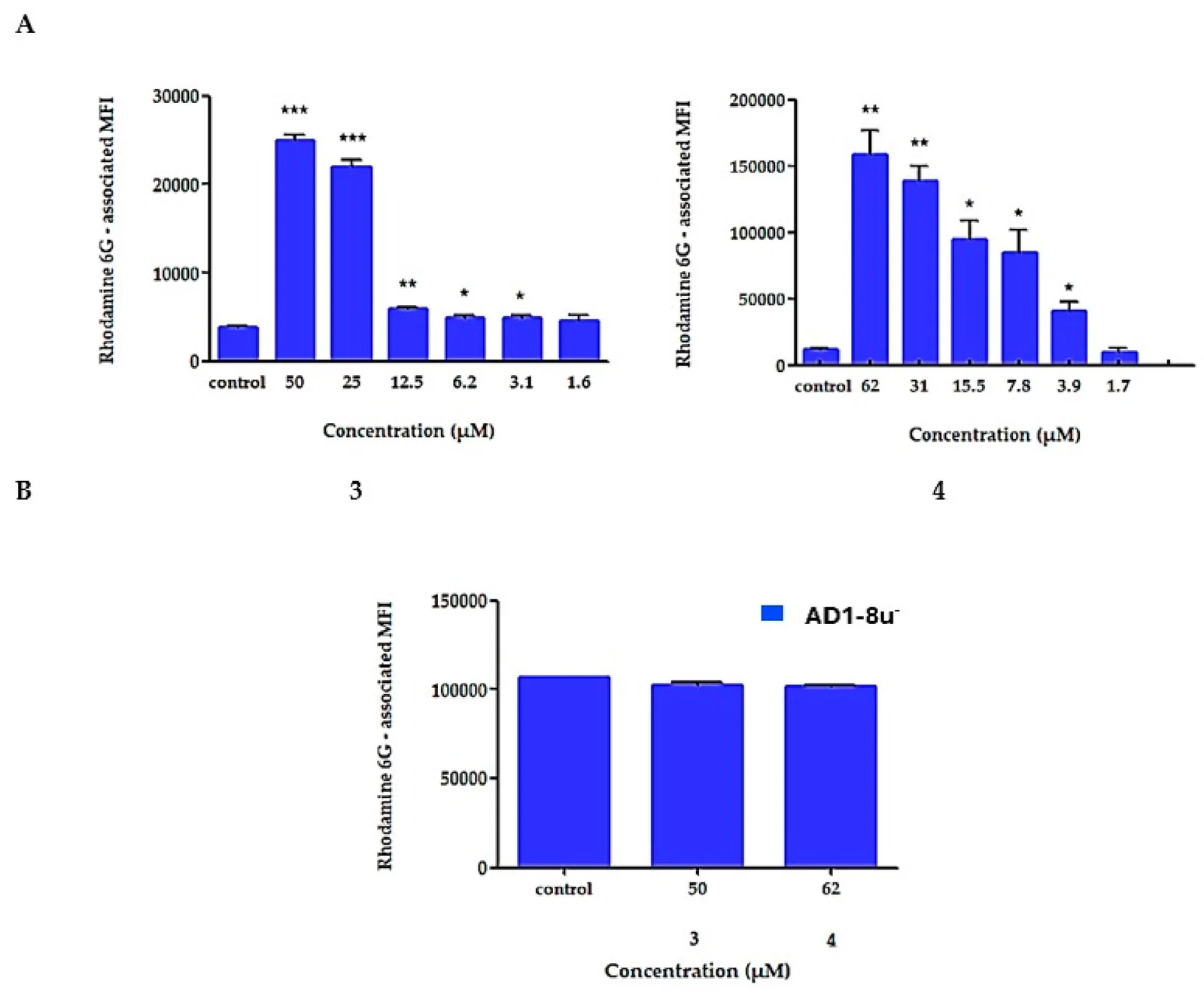
Effect of compounds **3** and **4** in (**A**) AD/CaCDR1 and (**B**) AD1-8u- cell lines assessed by the rhodamine 6G accumulation assay. Significant differences from the control were determined using a one-tailed paired Student’s *t*-test (*** *p* < 0.001; ** *p* < 0.01; * *p* < 0.05).

To validate the results obtained in the Nile red accumulation assay, the modulatory activity of the most active compounds, **3** and **4**, was further evaluated using rhodamine 6G, a fluorescent substrate transported by the Cdr1 efflux pump. Both compounds effectively reduced rhodamine 6G efflux, as evidenced by increased intracellular fluorescence intensity (MFI) of the dye in AD/CaCDR1 cells, with low MEC values of 3.1 µM and 3.9 µM for compounds **3** and **4**, respectively (*p* < 0.05) (Table 6, Figure 5A and Figure S11). Compared with the positive control, FK506 at 20 µM (FIF = 1.73 ± 0.24), compounds **3** and **4** showed stronger activity, as indicated by higher MFI values and lower MEC values (*p* < 0.05). Furthermore, neither compound affected rhodamine 6G accumulation in the AD1-8u^−^ null mutant strain, whereas effects were observed in the Cdr1-overexpressing strain. (Figure 5B).

### 2.5. Molecular Modeling of Compounds Isolated from Solanum atriplicifolium

Molecular modeling studies were performed to rationalize the preferential Cdr1 inhibitory activity of compounds **3** and **4** and to characterize the binding interactions responsible for their stabilization within substrate-binding pocket of the transporter. To this end, Cdr1 complexes with compounds **1**–**4**, fluconazole, the fluorescent substrates Nile red and rhodamine 6G, and the reference inhibitor milbemycin oxime (co-crystallized with the transporter and used as the positive control for the in silico studies) were prepared for molecular dynamics (MD) simulations.

***Ligand preparation***. The mutarotation equilibrium was first evaluated for the glycosides, all of which possess a free reducing anomeric hydroxyl group. Geometry optimizations and free-energy calculations were performed at the DFT level using the CAM-B3LYP/6-31G(d,p) method with an implicit water solvent model. The calculations indicated that compounds **1** and **4** predominantly adopt the β-anomeric form with populations of 80% and 92%, respectively. Compound **3** exists as an approximately equimolar mixture of α- and β-anomers (49% and 51%, respectively), whereas compound **2** shows a moderate preference for the α-anomer (59% α and 41% β). The calculated Gibbs free energies and the corresponding Boltzmann populations at 300 K are summarized in Table 7. Absolute electronic energies, zero-point energy corrections, and thermal contributions to the Gibbs free energy are provided in the Supplementary Material, Table S1. The structures of fluconazole, Nile red, and rhodamine 6G were also optimized at the same level of theory.

***Molecular Docking***. The subject compounds and the reference substrates and the inhibitor were docked throughout the entire transmembrane region of Cdr1, as no prior assumptions were made regarding the location of the binding site. AutoDock 4.2.6 and AutoDock Vina were first evaluated for their ability to reproduce the experimental binding pose of fluconazole. AutoDock Vina provided the closest agreement with the cryo-EM structure and was therefore selected for docking the complete set of ligands. Although compounds **1**–**4** exhibited more favorable docking scores than fluconazole, the resulting docking poses were used only as starting conformations for the molecular dynamics (MD) simulations. Because docking scores for structurally diverse, highly flexible, and polar ligands binding within large transmembrane cavities generally have limited discriminative power, MD-based analyses were subsequently employed to better assess and differentiate the binding affinities of the ligand series.

***Molecular Dynamics simulations and free energies of binding (ΔG°_b_)***. All protein–ligand complexes were subjected to 100–130 ns. The duration of each simulation was determined based on stabilization of the ligand root-mean-square deviation (RMSD), with representative RMSD plots provided in the Supplementary Material, Figure S12. Trajectory analyses showed that the dominant binding mode was maintained throughout the simulations for all active compounds. Cluster analysis further confirmed that the most populated conformational state corresponded to ligand binding within the crystallographic substrate-binding pocket, the same site in which fluconazole has been experimentally observed. Binding free energies were estimated using both MM-GBSA and MM-PBSA analyses of the equilibrated MD trajectories. For each ligand, the reported values correspond to the average of two to four independent simulations and are summarized in Table 8.

**Figure 6:**
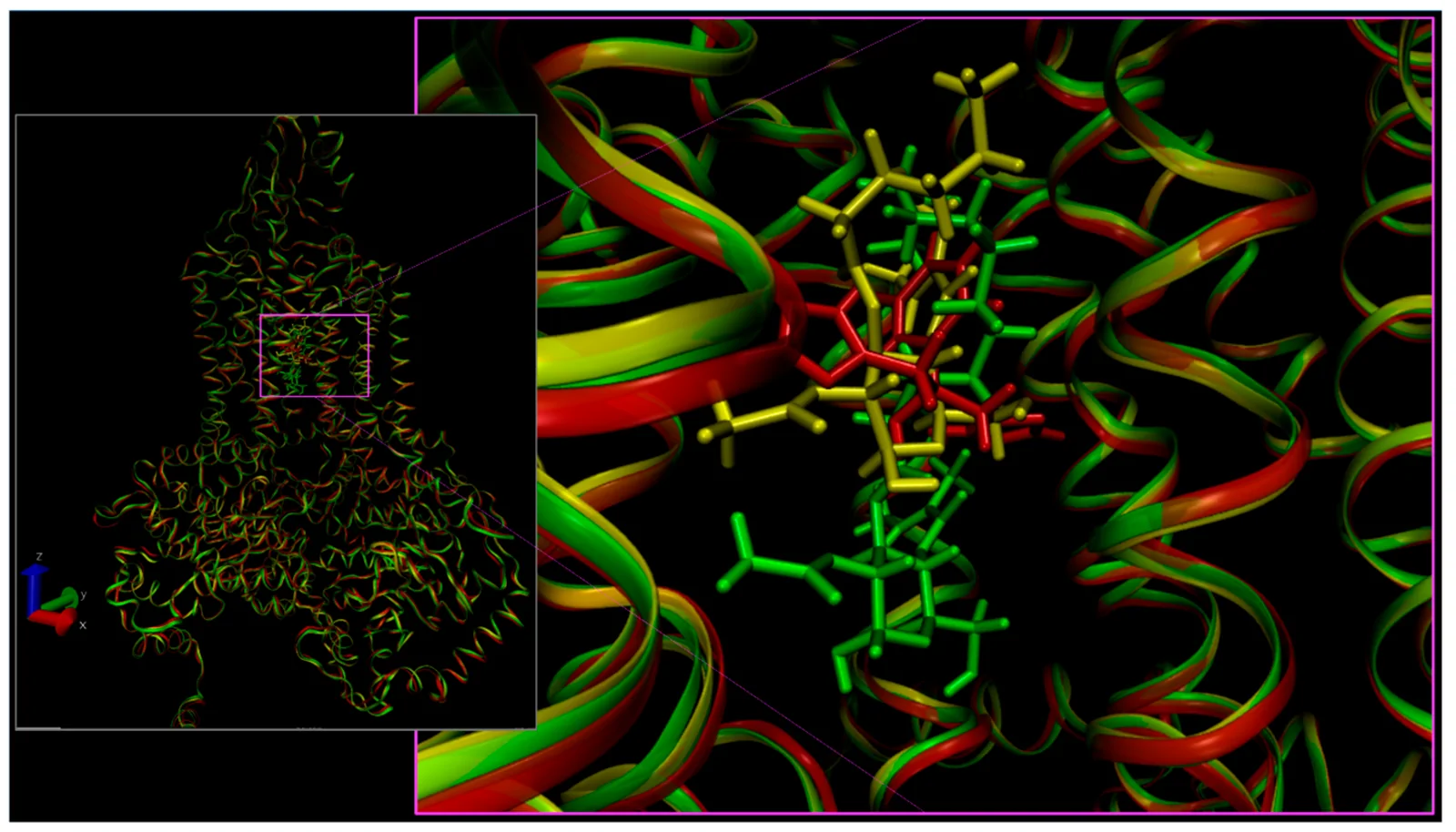
Superimposition of the structures of the most populated cluster (from the last 90 ns of simulations cluster analysis) for compound **3** (α-anomer) (green), compound **4** (β-anomer) (yellow) and fluconazole (red).

**Figure 7:**
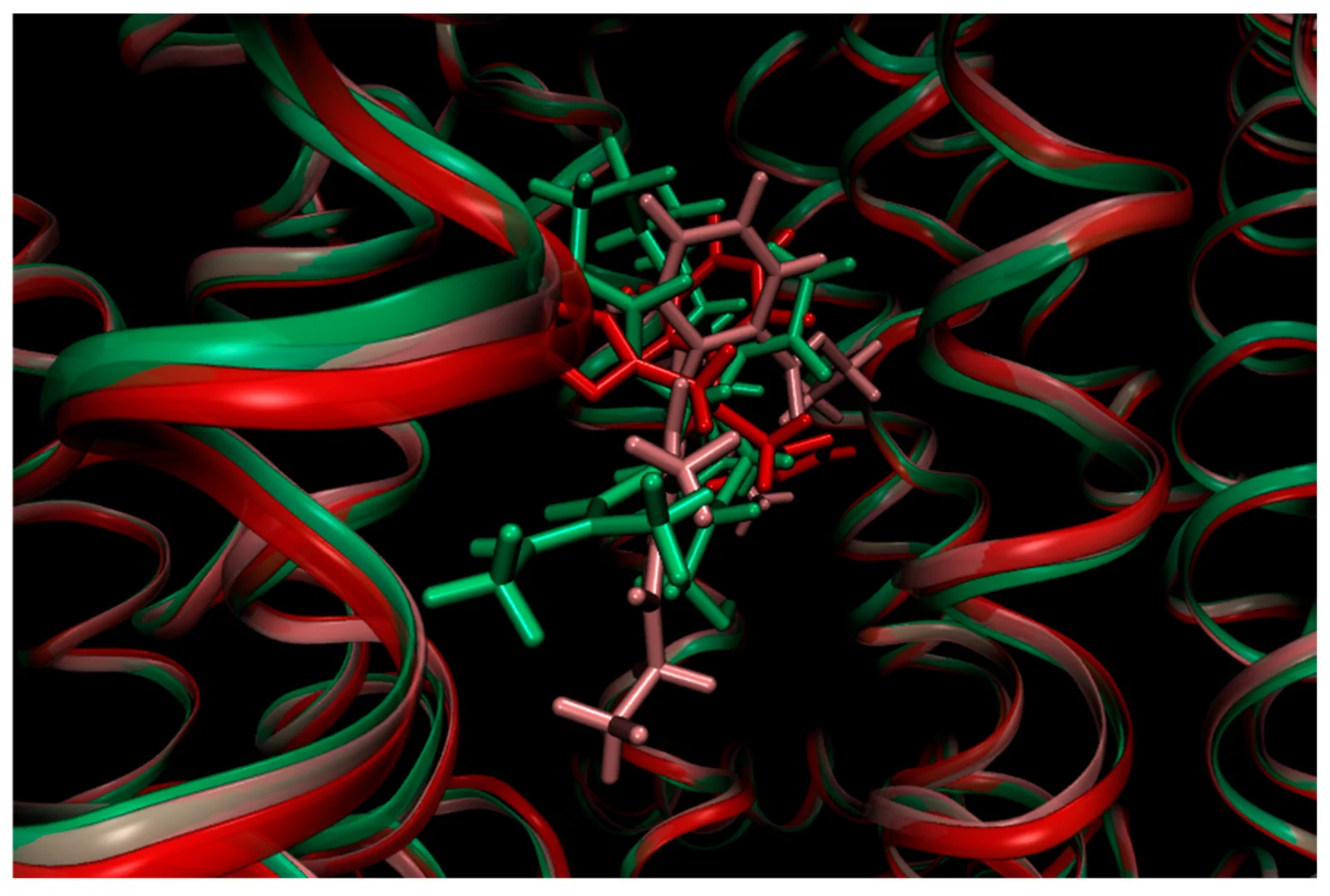
Superimposition of the representative structures of the most populated clusters for fluconazole (red), compound **3** (β-anomer, green) and rhodamine 6G (pink). The view is the same as in Figure 6.

The calculated ΔG°_b_ values revealed that the most active compounds **3** and **4**, exhibit comparable or even higher affinity for Cdr1 than the reference inhibitor, milbemycin oxime and significantly more favorable binding energies than those obtained for the fluorescent dyes and for fluconazole. The binding poses of a representative structure from the most populated cluster of the trajectory are superimposed in Figure 6 for compounds **3**, **4**, and fluconazole. Since the binding modes of compounds **3** and **4** clearly overlap with that of fluconazole and their binding free energies are more negative, it can be inferred that they would compete with this agent for the same binding site. Similar superimposition analyses were also performed for the reference inhibitor milbemycin oxime (Supplementary Material Figures S13 and S14). Interestingly, the binding poses of the fluorescent dyes also occupy the same pocket, as shown in Figure 7 (see also Supplementary Material Figure S15), providing an in silico rationale for their use in accumulation assays, in addition to the advantage of their high fluorescence quantum yield.

**Figure 8:**
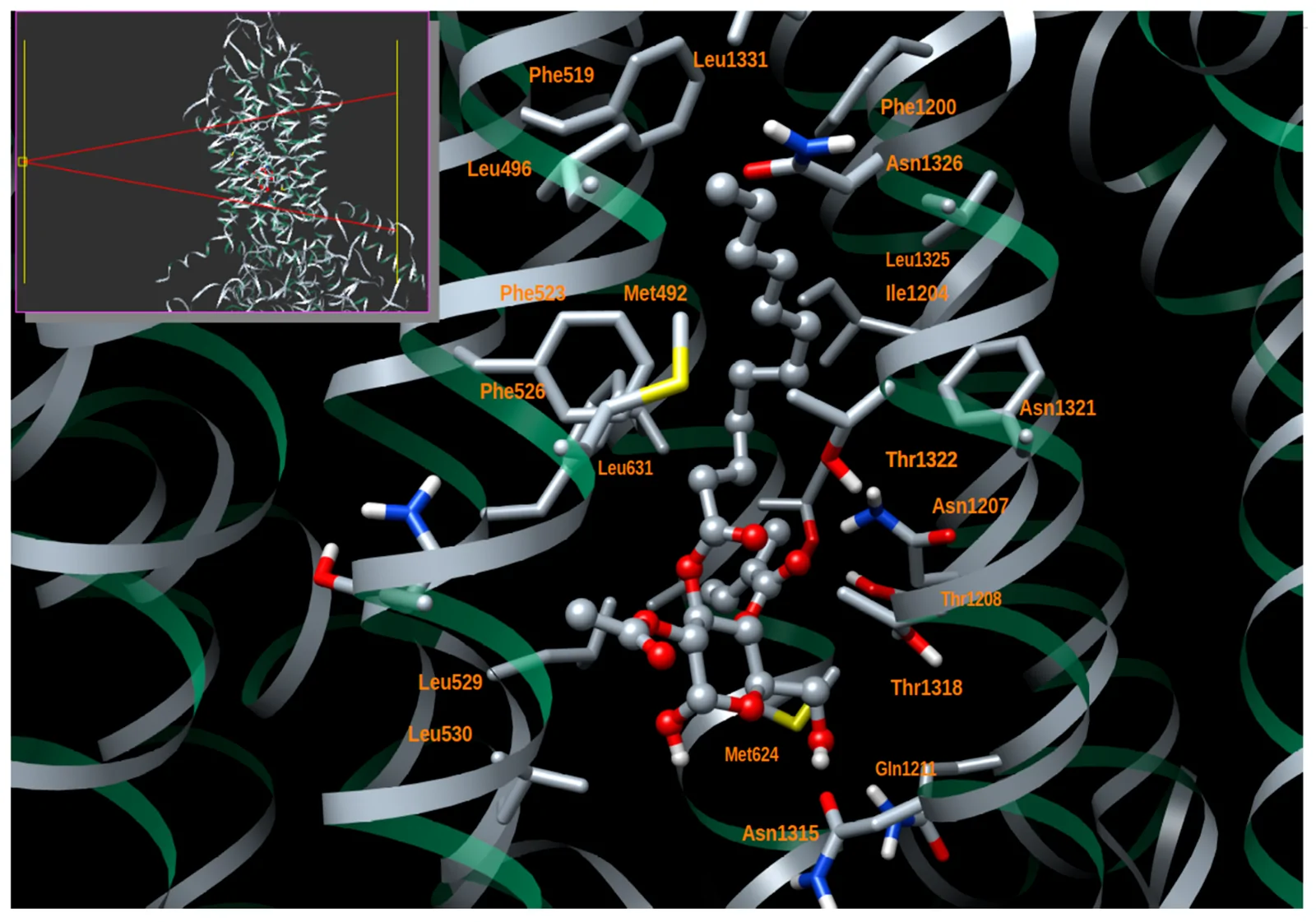
Representative structure from the most populated cluster of the trajectory for compound **3**α. The upper-left inset shows the orientation of the view.

**Figure 9:**
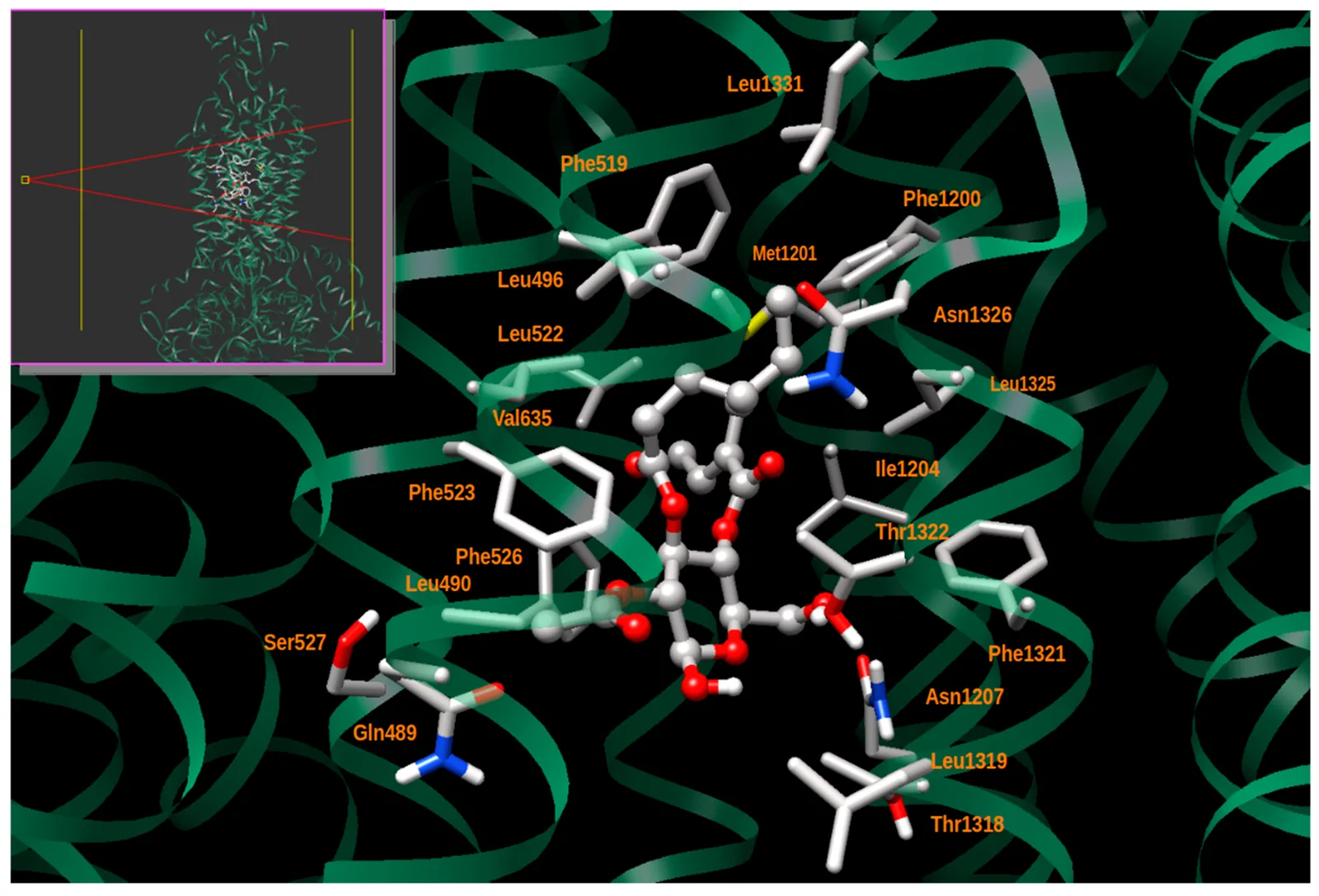
Representative structure from the most populated cluster of the trajectory for compound **4**β. The upper-left inset shows the viewing orientation.

**Figure 10:**
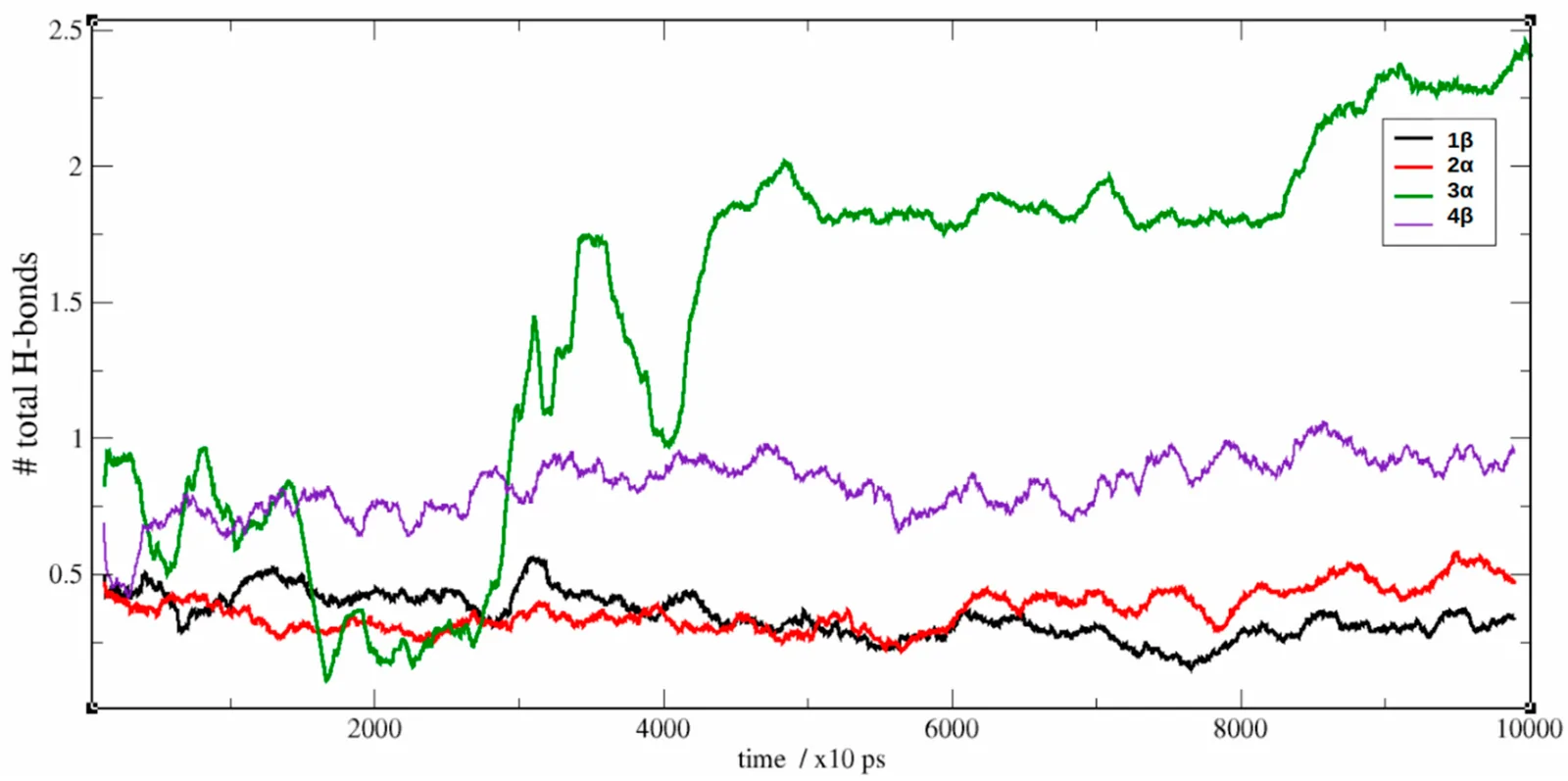
Overall number of persistent H-bonds along the trajectory with running average of 250 frames in order to reduce the noise.

The binding poses of the most active compounds **3** and **4**, are shown in detail in Figure 8 and Figure 9, respectively, based on representative snapshots from the most populated cluster of each trajectory. The two active compounds engaged distinct yet complementary intermolecular polar interaction networks within the binding site. Compound **3**α establishes the most extensive hydrogen-bond network in the series, forming contacts with ASN1240, GLN1244, ASN1348, and THR1351 through interactions involving both the anomeric hydroxyl and the free C-6 hydroxymethyl moiety. GLN1244 and ASN1348 are contacted exclusively by compound **3α** among all evaluated ligands, suggesting that the dodecanoyl chain at C-3, the longest acyl substituent in the series, positions the sugar core in a geometry that uniquely enabled the accesses to these residues. Compound **4**β, which bears a shorter hexanoyl chain at C-3, forms a more focused network with ASN1240 and THR1355, consistent with strong hydrogen bonds. In contrast, compounds **1** and **2** establish fewer intermolecular polar contacts, lack the persistent intramolecular hydrogen bond, and in several forms interact with MET657, a residue not contacted by the active compounds. This suggests a subtly different binding geometry that is less favorable for transporter modulation, despite the relatively small differences in ΔG°_b_ values. The overall persistence of the total number of H-bonds along the whole trajectories is compared in Figure 10 for compounds **3** and **4** versus **1** and **2** based on four representative simulations. In general, the most active species maintain a higher and more sustained number of hydrogen bonds over time.

**Figure 11:**
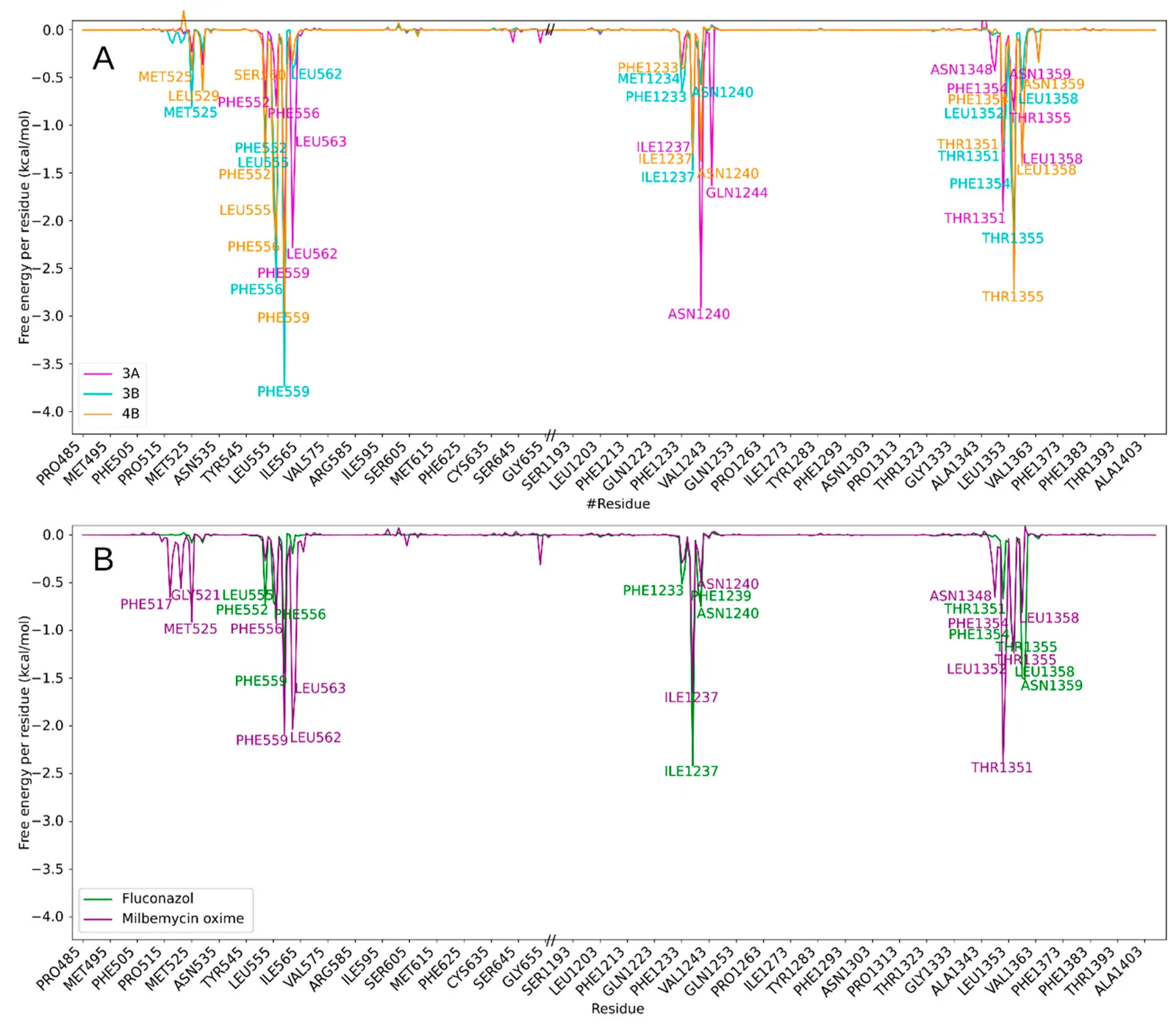
Per-residue MM-GBSA energy decomposition for compounds **3α**, **3β**, and **4β** (**A**) and the reference ligands fluconazole and milbemycin oxime (**B**) in complex with Cdr1. Stabilizing contributions (negative contribution to ΔG°_b_) from individual residues are shown for all five ligands. Only residues from position 485 onward are displayed, as no significant contributions were observed in the preceding sequence. Residues exceeding the labeling threshold are indicated. Discontinuous in the x-axis (//) indicate regions with negligible contributions that were omitted for clarity.

The per-residue energy decomposition profiles for compounds **3**α, **3β** and **4**α are shown in Figure 11A) in comparison with fluconazole and the reference inhibitor milbemycin oxime (Figure 11B). Profiles for the remaining compounds, dyes and reference ligands are provided in the Supplementary Material Figures S16–S18. Both active compounds engaged a conserved set of residues spanning the two hydrophobic clusters of the transmembrane cavity (PHE552–LEU562 and ILE1237–LEU1358), with additional polar contributions from ASN1240, THR1351, THR1355, and PHE1354. The interaction profile of compound **3α** is further characterized by additional contributions from GLN1244 and ASN1348, consistent with the more extensive hydrogen-bond network and the higher binding free energy observed for this compound.

A notable feature of the per-residue energy contribution patterns is that the interaction peaks observed for fluconazole are largely reproduced by compounds **3** and **4**, with additional contributions unique to the studied ligands. Moreover, residues that contribute to binding of the reference inhibitor (but are not involved in fluconazole binding) are also engaged by the compounds investigated here, suggesting an overlap with multiple experimentally validated binding modes within the transporter cavity.

The setup for the long molecular dynamics simulations described here is sufficiently reliable to capture most of the induced-fit effects involving the side chains of contacting residues, thereby providing valuable insights into the dynamics of protein–ligand interactions. However, certain subtle effects, particularly long-range conformational changes in the α-helical TMD barrel induced by different ligands, may be underestimated in the current MD setup. This limitation arises from the implicit membrane treatment, which employs harmonic restraints on backbone atoms to maintain the transmembrane domain structure close to the reference conformation. Although this approach has proven reliable and successful for numerous transmembrane proteins, particularly P-gp, an ABC transporter closely related to Cdr1p [30,31,32], we include here preliminary results from larger-scale MD simulations of Cdr1p embedded in a realistic lipid bilayer. The lipid composition, pH, and ionic strength of this membrane closely mimic the physiological conditions of the *C. albicans* plasma membrane [33] (see Section 3 and Supplementary Material for details).

For the full-system simulations (protein/ligand/lipids/ions/water), we selected fluconazole (the therapeutic substrate) and the best test compound among those simulated, designated **3α**. These simulations served a dual purpose: (i) to verify that our best test compounds effectively compete with fluconazole for the same binding site and significantly outperform it in terms of ΔG°_b_; and (ii) to confirm that the main residue–ligand contacts and their contributions to overall complex stabilization are reasonably maintained under more realistic membrane conditions [34].

**Figure 12:**
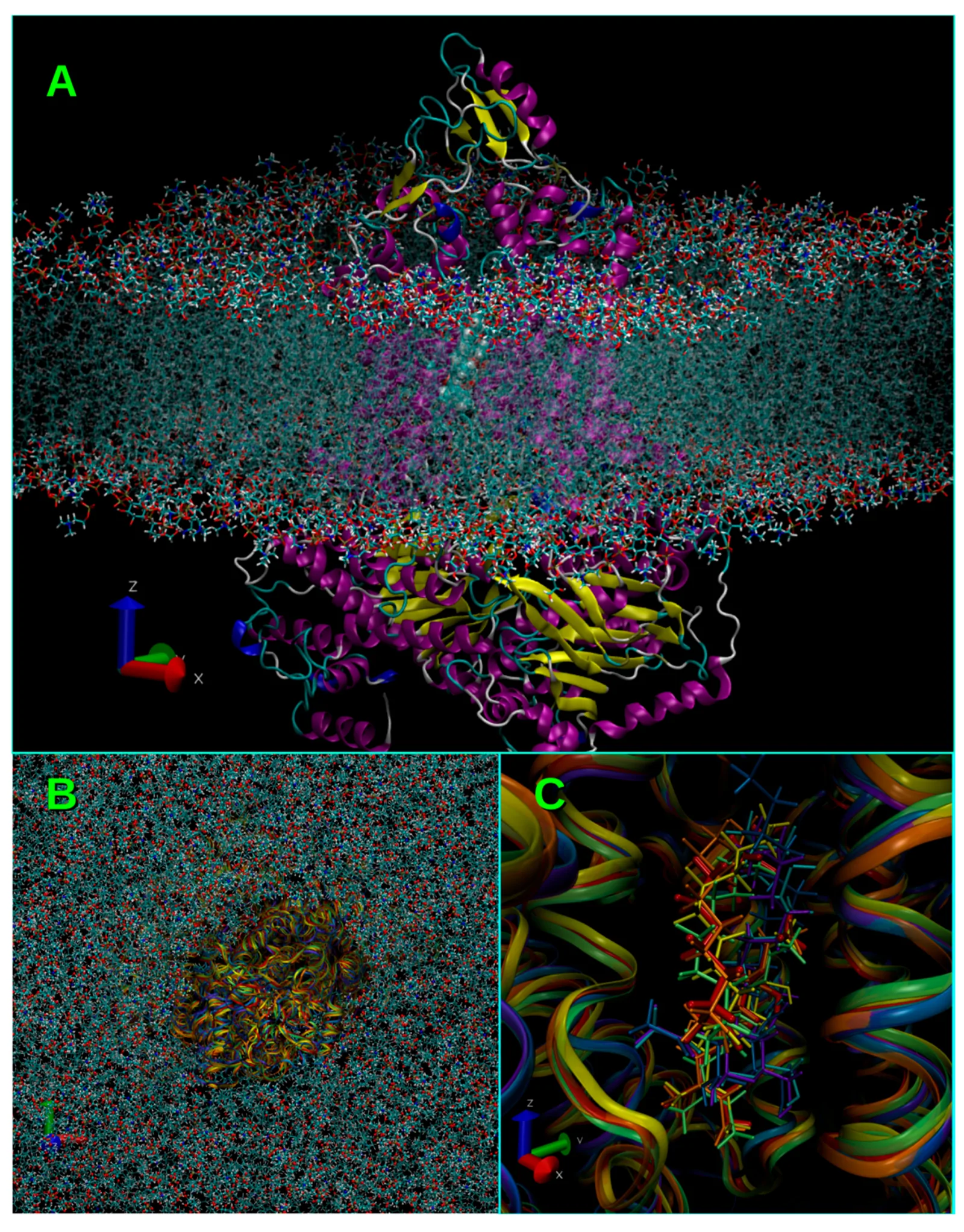
(**A**) Snapshot of the most populated cluster from the 100 ns molecular dynamics trajectory. Cdr1 is shown as a cartoon, compound **3**α as VdW spheres, the polar heads of the lipids as CPK and the hydrophobic lipid chains and ergosterol molecules as semi-transparent licorice; water and ions are not shown. (**B**,**C**) Two views of the superimposition of six snapshots for the Cdr1/3α complex. Rainbow colors range from red to yellow for the first clusters of the molecular dynamics simulation of the full system containing lipids and from green to violet the general implicit membrane setup; lipids, water and ions are not shown.

The simulation setup for compound **3α** is illustrated in Figure 12A, with analogous views and further details for fluconazole provided in Supplementary Material Figures S19–S21. Superimposition of three snapshots taken from the most populated cluster (last 50 ns) of both the implicit-membrane and realistic-membrane simulations is shown in Figure 12B,C, revealing that similar binding poses are sampled throughout the simulations. The per-residue contributions to complex stabilization are compared between the two simulation types in Supplementary Material Figure S22. These comparisons indicate that, on average over the last equilibrated trajectories, the contributions of the main residues discussed above are comparable between the two simulation series. In agreement with the findings from the simpler implicit-membrane simulations, the poses of **3α** and fluconazole clearly overlap throughout the simulation time (Supplementary Figures S20 and S22). However, as previously observed, the test compound **3α** exhibits a substantially more favorable ΔG°_b_ than fluconazole, with values of –32.4 vs. –19.5 kcal/mol, respectively. While the good agreement in the principal binding dynamics features between the two approaches for the two selected compounds does not permit direct extrapolation to the whole series, these simulations nonetheless provide supportive (though not fully confirmatory) validation of the simpler method employed for the complete compound set.

Collectively, these results support a model in which the superior Cdr1 modulatory activity of compounds **3** and **4** arises from the convergence of three structural features: the free C-6 hydroxymethyl group, which enables an intramolecular hydrogen bond that preorganizes the α-glucopyranose core; the acyl chain length at C-3, which positions the scaffold to engage key polar residues within the binding site; and the resulting intermolecular hydrogen-bond network involving ASN1240, THR1351, THR1355, GLN1244, and ASN1348, which complements the hydrophobic packing provided by the acyl chains within the transmembrane cavity. In contrast, blocking the C-6 position in compounds **1** and **2** appears sufficient to disrupt this binding mode resulting in reduced potency and decreased affinity for Cdr1 binding.

## 3. Materials and Methods

### 3.1. Materials and Chemicals

Nile Red and rhodamine 6G were purchased from Sigma-Aldrich (St. Louis, MI, USA). The antifungal agent fluconazole (FCZ, purity 98.5%) was obtained from Parafarm (Buenos Aires, Argentina). FK506 (tacrolimus, purity ≥ 98%) was purchased from Carbosynth Ltd (Compton, Berkshire, UK). Sterile plastic laboratory supplies were purchased from Greiner Bio-One (Frickenhausen, Germany). All solvents used were HPLC grade. Mass spectrometry (HR-ESIMS) was performed on a Bruker micrOTOF-Q II (Bruker Corporation, Ettlingen, Germany) coupled to an Agilent RRLC 1200 ultra-high-performance chromatograph (Agilent Technologies, Inc., Santa Clara, CA, USA) using positive mode electrospray ionization. One-dimensional and two-dimensional NMR spectra were recorded with a Bruker AVANCEII 400 and Bruker AVANCEIII 600 HD spectrometer (Bruker Corporation, Germany). HPLC was performed on a Shimadzu LC-10 AS (Shimadzu Corp., Tokyo, Japan), equipped with a Phenomenex Prodigy 5 μ ODS reverse-phase column (4.6 mm inner diameter × 250 mm). Flow cytometry was performed on a Life Technologies Attune-NxT flow cytometer with a 96-well automatic sampler (Thermo Fisher Scientific, Waltham, MA, USA).

### 3.2. Plant Material and Preparation of Extract

The aerial parts of *S. atriplicifolium* were collected in the mountainous region of Córdoba Province, Argentina, between December and March, −31.5861 latitude and −64.4581 longitude. Botanical identification was carried out by agricultural engineer G. Ruiz, who classified the specimen and assigned a voucher number (UCCOR 175), which was deposited in the Marcelino Sayago herbarium at the Faculty of Agricultural Sciences, Catholic University of Córdoba. The plant material was air-dried at room temperature for two weeks and then ground using a mechanical grinder. The extract was obtained by maceration of the powdered plant material with 96% ethanol (solid–solvent ratio 1:3, *w*/*v*) for 48 h, followed by filtration and evaporation to dryness under reduced pressure. The resulting extracts were stored in amber bottles, protected from light, at 4 °C. The extraction yield, expressed as a percentage relative to the weight of the air-dried plant material, was 18.59%.

### 3.3. Strains and Culture Conditions

Two clinical isolates obtained from patients at the University Hospital of the Federal University of Juiz de Fora, Minas Gerais, Brazil, were used. A *C. albicans* strain overexpressing CaMdr1 and CaCdr1 was designated as strain 1114 [18] and a *C. glabrata* strain overexpressing CgCdr1 was designated as strin 109 [5,35]. Both isolates were identified by MALDI-TOF mass spectrometry [5,35]. The minimum inhibitory concentration (MIC) was determined using the agar dilution method described below. Strain 1114 exhibited a 515-fold higher resistance to FCZ compared with the susceptible reference strain *C. albicans* ATCC 90028 (MIC_FCZ_ = 500 and 0.97 µg/mL, respectively), whereas strain 109 showed a 64-fold increase in resistance relative to *C. glabrata* ATCC 2001 (MIC_FCZ_ = 1000 and 15.6 µg/mL, respectively), in accordance with susceptibility breakpoints reported by CLSI (2020) [36]. ATCC strains were included as controls in all assays.

In addition, *S. cerevisiae* strains overexpressing specific transporters as well as a susceptible null mutant strain were used [23]. The null mutant strain (AD1-8u^−^), which lacks the genes encoding MDR efflux pumps showed an MIC_FCZ_ value of 2.0 μg/mL. The FCZ-resistant strain AD/CaCDR1 selectively overexpresses the *C. albicans* ABC transporter Cdr1 and showed an MIC_FCZ_ value of 250 μg/mL, whereas the FCZ-resistant strain AD/CaMDR1 selectively overexpresses the *C. albicans* MFS transporter Mdr1 (MIC_FCZ_ = 31.2 μg/mL). These *S. cerevisiae* strains were kindly provided by Dr. R. D. Cannon from the University of Otago, New Zealand.

All yeast strains were stored at −20 °C in Sabouraud dextrose broth (SDB; Difco Laboratories, Detroit, MI, USA) supplemented with 10% glycerol. Prior to use, strains were subcultured on Sabouraud agar (Laboratorio Britania, Buenos Aires, Argentina) or in SDB at 30 °C for 24 h. Cells from agar plates were used to prepare working suspensions in sterile saline solution.

### 3.4. Bioguided Isolation and Characterization of Compounds

The extract of *S. atriplicifolium* was selected for bioassay-guided isolation in order to identify the compound(s) responsible for this activity. This selection was based on its lack of intrinsic antifungal activity against all tested yeast strains, while efficiently inhibiting both efflux transporters, Cdr1 and Mdr1 [23]. The latter effect was supported by the observed MEC values of 25 μg/mL against *C. albicans* 1114 and *C. glabrata* 109, resulting in up to a fourfold reversal of FCZ resistance [23]. Similarly, MEC values of 100 μg/mL were recorded for the AD/CaMDR1 and AD/CaCDR1 strains, also resulting in a fourfold reduction in resistance [23]. The ethanol extract of *S. atriplicifolium* (16 g) was subjected to vacuum liquid chromatography on silica gel (300 g, 70–230 mesh, 11.0 × 24.0 cm; Macherey and Nagel, Düren, Germany), using a gradient of increasing polarity of hexane (Hex)/ethyl acetate (EtOAc)/methanol (MeOH). This procedure yielded eighteen fractions, which were subsequently combined into nine pooled fractions (F1–F9) (Supplementary Material, Figure S23) based on their thin-layer chromatography (TLC) profiles (silica gel 60 F254, Merck; mobile phase Hex/EtOAc 30:70). The ability of these fractions to reverse FCZ resistance was evaluated by the agar reversion assay against *C. albicans* 1114, *C. glabrata* 109 (CgCdr1), AD/CaMDR1 and AD/CaCDR1, and corresponding sensitive strains. Fractions F1 and F2 showed activity at 25 μg/mL and were therefore further fractionated by silica gel column chromatography (6 g) (180 g, 230–400 mesh, 3.0 × 60 cm), eluting with a Hex/EtOAc/MeOH gradient of increasing polarity. This step yielded five subfractions (F_1,2_1-F_1,2_5) as determined by TLC analysis (mobile phase Hex/EtOAc 80:20). Among these, fractions F_1,2_2 and F_1,2_3 efficiently reverse FCZ resistance at 12.5 μg/mL and were subsequently subjected to a second silica gel column (3 g) (90 g, 230–400 mesh, 1.5 × 45 cm) under similar elution conditions. This separation produced eight subfractions (F_1,2.2,3_1-F_1,2.2,3_8) of which F_1,2.2,3_6 and F_1,2.2,3_7 were active at 6.25 μg/mL. These active fractions were further purified using a Sephadex LH20 column (3 × 40 cm) with MeOH as the mobile phase, yielding 7 fractions (F_1,2.2,3.6,7_1-F_1,2.2,3.6,7_7) based on TLC profile (mobile phase Hex/EtOAc 50:50). Fractions F_1,2.2,3.6,7_1 and F_1,2.2,3.6,7_2 retain activity at 6.25 μg/mL and were therefore selected for further purification by flash chromatography using an Isolera system (Biotage, Uppsala, Sweden) equipped with a Biotage SNAP Ultra reverse phase C18 cartridge (12 g of silica). Elution was performed with a mixture of ACN/water 65:35 at a flow rate of 10 mL/min yielding four fractions. The activity of these fractions was evaluated, revealing that F_1,2.2,3.6,7.1,2_1 was active at 6.25 μg/mL, F_1,2.2,3.6,7.1,2_2 was weakly active at 100 μg/mL, and F_1,2.2,3.6,7.1,2_3 and F_1,2.2,3.6,7.1,2_4 were inactive (>100 μg/mL). Further analysis by HPLC was performed using an isocratic mobile phase of ACN/water (70:30), with detection at 190 nm and a flow rate of 1 mL/min. Fraction F_1,2.2,3.6,7.1,2_1 yielded four major compounds. The peaks at retention times of 5.2, 6.9, 10.0, and 11.8 min corresponded to compounds **1**–**4**, respectively (18.4, 16.9, 14.8 and 15.9 mg, respectively). In addition, fraction F_1,2.2,3.6,7.1,2_2, yielded compound **5** (18.8 mg) with a retention time at 23 min, while from F_1,2.2,3.6,7.1,2_3 yielded compound **6** (17.7 mg) with a retention time of 29 min.

The chemical structure of the six isolated compounds **1**–**6** (Figure 1) was elucidated by NMR spectroscopy.

### 3.5. Fluconazole Chemosensitization Assay on Agar

The ability of compounds **1**–**6** to chemosensitize the AD/CaCDR1 and AD/CaMDR1 strains to FCZ was evaluated using the antifungal susceptibility protocol described previously [23]. Briefly, the compounds were tested at a fixed concentration of 100 μM in combination with FCZ at 62.5 μg/mL and 7.8 μg/mL, corresponding to one-quarter of the MIC values for AD/CaCDR1 and AD/CaMDR1, respectively. Compounds that exhibited sensitizing activity at this initial concentration were subsequently evaluated using serial dilutions to determine their MEC values in the presence of FCZ at one-quarter of its MIC. The MEC was defined as the lowest concentration of each compound that, in combination with a subinhibitory concentration of FCZ (one-quarter of its MIC), resulted in complete absence of visible yeast growth, whereas FCZ alone at this concentration did not inhibit growth. In parallel, the FCZ-sensitive AD1-8u^−^ strain was included to assess potential synergistic effects unrelated to efflux transporter involvement. For this purpose, compounds **1**–**6** were tested at 100 μM in combination with FCZ at 0.50 μg/mL, corresponding to one-quarter of the MIC for this strain. It is important to note that, at 100 μM, none of the compounds affected the viability of the three assayed strains. FK506, a known Cdr1 inhibitor [37], was used as a positive control, whereas 5% ethanol served as the negative control.

### 3.6. Nile Red Intracellular Accumulation Assay

The Nile red accumulation assay was performed to evaluate the ability of the most active compounds **1**–**4** to promote intracellular accumulation of the dye, which is a known substrate of both efflux transporters Cdr1 and Mdr1 [5,38,39]. Both resistant and sensitive yeast strains were cultured in Sabouraud broth for 24 h to reach the exponential growth phase. Cells were then harvested by centrifugation, washed twice with distilled water, and incubated on ice for 2 h. Subsequently, cells were adjusted to a density of 1 × 10^6^ cells/well and incubated in 96-well U-bottom microtiter plates containing Sabouraud broth, in the absence or presence of the tested compounds, dissolved in PBS, at a maximum concentration of 25 μg/mL. Plates were incubated for 2 h at 30 °C with shaking at 200 rpm. Nile red was then added to each well at a final concentration of 7 μM. For Cdr1 assays, 2% glucose was included, and plates were further incubated for 1.5 h under the same conditions. After incubation, cells were washed twice with PBS and resuspended in DMSO for fluorescence measurement. Cells treated with FK506 (20 and 10 μM) were included as a positive control, while cells treated with 1% (v/v) DMSO served as the negative control.

Fluorescence intensity was measured for 25,000 cells using a Life Technologies Attune-NxT flow cytometer equipped with a 96-well autosampler (Thermo Fisher Scientific, Waltham, MA, USA). Excitation was performed with a 488 nm laser, and emission was collected using a 574/26 nm filter.The mean fluorescence intensity (MFI) was analyzed using FlowJo V10 software (Tree Star Inc., Ashland, OR, USA). Efflux pump inhibition was determined using the fluorescence intensity factor (FIF), calculated as the ratio between the MFI of Nile red in the presence of the inhibitor and the MFI of Nile red alone. MFI values obtained for the negative control with DMSO were comparable to those of Nile red alone.

### 3.7. Intracellular Accumulation Assay with Rhodamine 6G

To confirm the results obtained in the intracellular accumulation assay using Nile red, the inhibitory activity of the most active compounds **3** and **4** was also evaluated using rhodamine 6G. Efflux pumps belonging to the ABC superfamily in FCZ-resistant *Candida* cells actively extrude this dye following its passive uptake; therefore, it is commonly used to assess the in vitro efficiency of potential efflux pump inhibitors [24,25]. For this assay, the same procedure described for the Nile red accumulation method was followed. Rhodamine 6G was added to a final concentration of 5 µM. Fluorescence was measured with excitation at 488 nm, and emission was collected using a 585/42 nm filter. The mean fluorescence intensity (MFI) was analyzed using FlowJo software, and the corresponding fluorescence intensity factors (FIFs) were calculated as above reported. MFI values of the DMSO negative control cells were comparable to those obtained with rhodamine 6G alone.

### 3.8. Molecular Modeling

Given that compounds **3** and **4** showed markedly stronger chemosensitizing activity against the AD/CaCDR1 strain than against AD/CaMDR1, together with a greater increase in Nile red and rhodamine 6G intracellular accumulation in Cdr1-overexpressing cells, molecular modeling studies were focused on the Cdr1 transporter. This approach aimed to rationalize the preferential interaction of the active acylglucoses with this efflux pump and to identify the molecular determinants underlying their inhibitory activity.

The structural model of *Candida albicans* Cdr1 was constructed starting from the experimentally resolved structure (PDB ID: 9IUL; [40], co-crystallized with fluconazole. Missing residues were modeled using the AlphaFold2-predicted structure corresponding to UniProt entry Q5ANA3 [41,42,43] followed by further local optimization and preparative relaxations. The 33 N-terminal residues present in the AlphaFold2 model but absent from the experimental structure were removed, as they do not form part of the crystallographically resolved protein and their inclusion would have increased computational cost without contributing structural information. The resulting structure was prepared and refined using tools from the AMBER24 software package [44] and visualized with PyMOL 2.5 [45] VMD 2.0 [46] and Chimera 1.17.

Compounds **1**–**4** were modeled considering both α and β anomeric forms, which were analyzed independently given their potential differences in conformational behavior and binding properties. The reference ligands fluconazole, rhodamine 6G, Nile red, and milbemycin oxime were included for comparative purposes. After a conformational search when necessary the geometry optimization was done using the density functional model CAM-B3LYP with the 6-31G(d,p) basis set using Gaussian 16 [47]. For compounds 1–4, the relative population of each anomer under equilibrium conditions were calculated in terms of relative free energy calculations at the same level of theory with implicit water as solvent. The free energies were obtained from the harmonic frequencies calculation and subsequent thermochemistry analylsis including zero point energy and thermal corrections to the Gibbs energy. The anomeric equilibrium constant (Keq) was derived from the free energy difference between the α and β forms according to the Boltzmann distribution at 298.15 K.

To validate the docking protocol, a re-docking procedure was performed using the co-crystallized ligand fluconazole. Two docking engines, AutoDock Vina [48] and AutoDock 4, ref. [49] were evaluated based on their ability to reproduce the experimental binding pose. of fluconazole. AutoDock Vina showed superior performance, then Vina was selected for all subsequent docking studies.

Docking simulations were carried out using AutoDock Vina, targeting the central substrate-binding cavity of Cdr1, considering the whole internal transmembrane domains. For each compound, the binding pose with the lowest predicted binding energy was selected for subsequent analyses.

The selected protein–ligand complexes were subjected to molecular dynamics (MD) simulations using the AMBER ff14SB force field [50] for the protein. Ligands were parameterized using the general AMBER force field (GAFF2) [51]. Partial atomic charges were assigned using the AM1-BCC method, except for charged ligands such as rhodamine 6G, for which RESP charges were derived from electrostatic potentials calculated at the quantum mechanical level using Gaussian 16. Each complex was solvated in an explicit TIP3P water box, and counterions were added to neutralize the system prior to MD simulations. Taking into consideration the transmembrane nature of Cdr1, a harmonic constraint of 50 kcal/Å2 was applied to the backbone atoms. This protocol and the general setup for molecular dynamics was as previously described and successfully applied to another ABC transporter, the human P-gp (ABCB1) [31,32,52]. Briefly, this include (I) 600 conjugated gradient minimization of the solvent and ions with the complex fixed, (II) 6500 conjugate gradient minimization steps of the whole system; (III) 100 ps slowly heating in the NTV ensemble with the protein positionally restrained in the backbone; (IV) 100–120 ns of simulation in the NTP ensemble, at 1 atm and 300 K. Procedures III-IV were repeated in two or three independent trajectories using the Andersen thermostat and barostat. The electrostatic interactions were computed using the Particle Mesh Ewald (PME) method with a cutoff of 10 Å. The SHAKE algorithm, as implemented in pmemd.cuda, was applied to constrain hydrogen-heavy atom bonds.

Trajectory analyses focused on the RMSD of the ligand and surrounding residues within 5 Å and binding mode stability throughout the simulation. To identify the most frequently visited binding states, cluster analysis was performed using UCSF Chimera 1.17 [53], grouping trajectory frames by ligand RMSD similarity. The most populated cluster, reflecting the conformational region most recurrently sampled by the ligand, was identified, and a representative 10 ns stable window from this cluster was selected for detailed characterization of persistent interactions within the binding pocket.

Binding free energies (ΔG°_b_) were estimated using MM-GBSA and MM-PBSA [54] over the last 10 ns of the stabilized MD trajectory. The informed ΔG°_b_ values were averaged over 2–4 independent trajectories. The per-residue energy decomposition analyses were additionally performed to identify amino acid residues contributing most significantly to ligand stabilization and to rationalize the experimentally observed differences in inhibitory activity among the evaluated acylglucoses.

For two selected cases, fluconazole and compound **3** (α-anomer), a fully realistic Cdr1/ligand system embedded in a *C. albicans* membrane was built. The structure of Cdr1p employed in the simulations corresponded to the model used in the molecular dynamics studies described above. From this structure, a lipid membrane was constructed using Packmol-Memgen [44], employing a representative mixture of the *C. albicans* plasma membrane composed of 1-palmitoyl-2-oleoyl-sn-glycero-3-phosphocholine (POPC), 1-palmitoyl-2-oleoyl-sn-glycero-3-phosphoethanolamine (POPE), ergosterol (ERG), 1-palmitoyl-2-oleoyl-sn-glycero-3-phospho-L-serine (POPS), 1-palmitoyl-2-oleoyl-sn-glycero-3-phospho-(1′-rac-glycerol) (POPG), 1-palmitoyl-2-oleoyl-sn-glycero-3-phosphate (POPA), and 1-palmitoyl-2-oleoyl-sn-glycero-3-phospho-myo-inositol (POPI), in a relative molar ratio of 36:29:12:9:6:6:14, respectively [33,34]. This composition was selected as a representative model of the fungal plasma membrane, incorporating the major phospholipid classes and the characteristic sterol of *C. albicans*.

Using Packmol-Memgen, the system was additionally solvated with TIP3P water molecules, and the necessary counterions were incorporated to neutralize the net charge of the system. Subsequently, parameterization was carried out using tleap, incorporating the force fields corresponding to the protein, lipid membrane, water, and ions, along with the parameters for fluconazole, which had been previously obtained using Antechamber 22.0 and its parameter library.

The initial relaxation of the system consisted of six successive energy minimization stages aimed at removing unfavorable contacts and relieving structural strains. First, a global minimization was performed using the steepest descent algorithm. Subsequently, two minimization stages of the water molecules and ions were carried out, while restraining the protein–ligand complex and the membrane. Next, two additional minimizations of the water and lipids were performed, keeping the protein–ligand complex restrained and progressively decreasing the restraint strength between the two stages. Finally, a full unrestrained minimization of the entire system was performed, allowing the simultaneous relaxation of all intra- and intermolecular interactions prior to the equilibration phase.

System equilibration was carried out gradually through simulations under NVT and NPT ensembles. Initially, an NVT stage was performed with harmonic restraints on the protein–ligand complex and a gradual heating from 0 to 100 K over 0.5 ns while maintaining a constant system volume. Subsequently, an NPT stage was performed, gradually increasing the temperature to 300 K and progressively reducing the restraints applied to the complex, allowing lipid rearrangement around the transporter and isotropic adjustment of the simulation box. These preparatory stages totaled 5 ns. Finally, a fully unrestrained NPT equilibration was performed for 100 ns at 310 K, allowing the coordinated stabilization of the protein, membrane, solvent, and ions before the start of the production simulation. During this stage, the protein, fluconazole, lipid membrane, water molecules, and ions evolved freely, allowing the evaluation of the structural stability of the complex and the interactions between Cdr1p, the ligand, and the lipid environment.

## 4. Statistical Analysis

Results are expressed as mean ± standard error (SE). Data were analyzed using one-tailed paired Student’s *t*-test, using GraphPad Prism 7.0. Values of *p* ≤ 0.05 were considered statistically significant. Experiments were performed in at least three independent replicates.

## Figures and Tables

**Table 1:** ^1^H and ^13^C chemical shift values for compounds **1** and **2** in methanol-*d*_4_ (δ in ppm and *J* values in Hz in parentheses).

		1				2			
Position	Type	δ_H_ (*J*)		δ_C_		δ_H_ (*J*)		δ_C_	
				(from HSQC and HMBC)				(from HSQC and HMBC)	
		*α*	*β*	*α*	*β*	*α*	*β*	*α*	*β*
1	CH	5.38, brs	4.43, d (5.2)	102.6	100.4	4.61, d (4.5)	4.43, dd (12.8, 5.0)	100.3	101.3
2	CH	4.80 ^a^, m	4.85 ^a^, m	71.3	71.8	4.88 ^a^, m	4.98, t (1.8)	71.9	71.4
3	CH	5.71, t (6.5)	5.46, t (10.4)	70.8	70.6	5.53, m	5.70, m	70.1	71.2
4	CH	5.36, dd (10.8, 6.6)	5.00, t (7.4)	71.6	70.7	5.45, t (6.8)	5.32, t (6.6)	70.8	73.5
5	CH	3.87, m	3.67, m	68.7	72.5	3.87, d (2.0)	3.60, m	67.8	69.5
6	CH	3.31, m	3.59, m	63.2	63.1	3.58, br. S	3.89, d (1.6)	63.1	63.1
1′	CO	-	-	170.9	170.9	-	-	170.9	170.9
2′	CH_3_	2.11, s	2.11, s	19.3	19.3	2.02, s	2.11, s	19.5	19.4
1″	CO	-	-			-	-		
2″	CH_2_	2.27, m	2.27, m	33.3	33.3	2.33, m	2.33, m	33.5	33.5
3″	CH_2_	1.67, m	1.67, m	24.7	24.7	1.61, m	1.61, m	24.4	24.4
4″	CH_2_	1.29, m	1.29, m	29.0	29.0	1.29, m	1.29, m	29.1	29.1
5″	CH_2_	1.29, m	1.29, m	29.0	29.0	1.29, m	1.29, m	29.1	29.1
6″	CH_2_	1.29, m	1.29, m	29.0	29.0	1.28, m	1.28, m	31.3	31.3
7″	CH_2_	1.29, m	1.29, m	29.0	29.0	1.32, m	1.32, m	22.1	22.1
8″	CH_2_	1.28, m	1.28, m	29.0	29.0				
8″	CH_3_					0.90, t (4.6)	0.90, t (4.6)	12.8	12.8
9″	CH_2_	1.32, m	1.32, m	22.0	22.0				
10″	CH_3_	0.90, t (4.6)	0.90, t (4.6)	12.7	12.7				
1‴	CO	-	-	167.1	167.1	-	-	167.1	167.1
2‴	C			128.1	128.1			128.1	128.1
3‴	CH	6.86, m	6.86, m	138.6	138.6	6.85, m	6.85, m	138.6	138.6
4‴	CH_3_	1.79, d (4.8)	1.79, d (4.8)	12.8	12.8	1.79, d (3.6)	1.79, d (3.6)	12.8	12.8
5‴	CH_3_	1.80, s	1.80, s	10.4	10.4	1.80, s	1.80, s	10.5	10.5
1″″	CO	-	-	176.1	176.1	-	-	175.9	175.9
2″″	CH	2.41, m	2.41, m	33.5	33.5	2.37, m	2.37, m	40.9	40.9
3″″	CH_3_	0.97, d (4.8)	0.97, d (4.8)	17.3	17.3				
3″″	CH_2_					1.48, m	1.48, m	26.2	26.2
4″″	CH_3_	1.03, d (4.0)	1.03, d (4.0)	17.1	17.1	0.94, t (2.4)	0.94, t (2.4)	10.3	10.3
5″″	CH_3_					1.00, d (4.4)	1.00, d (4.4)	15.3	15.3

^a^ Obtained from HSQC. Overlapping signal with deuterated methanol.

**Table 2:** ^1^H and ^13^C chemical shift values for compounds **3** and **4** in methanol-*d*_4_ (δ in ppm and *J* values in Hz in parentheses).

		3				4			
Position	Type	δ_H_ (*J*)		δ_C_		δ_H_ (*J*)		δ_C_	
				(from HSQC and HMBC)				(from HSQC and HMBC)	
		*α*	*β*	*α*	*β*	*α*	*β*	*α*	*β*
1	CH	4.60, d (2.8)	4.54, d (4.4)	99.8	100.5	4.61, d (2.8)	4.55, d (4.4)	99.5	100.2
2	CH	4.88 ^a^, d (18.4)	4.96, m	72.3	69.2	4.88 ^a^	4.66, t (4.2)	71.7	73.9
3	CH	5.29, m	5.22, t (6.4)	73.9	73.1	5.24, dd (11.2, 6.4)		73.3	
4	CH	5.00, dd (2.8, 1.8)	5.02, m	72.1	70.6	5.00, m	5.06, m	71.8	70.3
5	CH	3.97, m	3.51, m	72.5	70.2	3.96, m	3.58, m	71.5	69.8
6	CH_2_	3.57, m	3.94, m	61.8	61.8	3.57, m	3.91, m	62.9	62.9
1′	CO	-	-	170.9	170.9	-	-	170.9	170.9
2′	CH_3_	2.06, s	2.11, s	19.7	19.5	2.01, s	2.11, s	19.8	19.7
1″	CO	-	-	173.1	173.1	-	-	173.1	173.1
2″	CH_2_	2.32, m	2.32, m	33.8	33.8	2.33, m	2.33, m	34.1	34.1
3″	CH_2_	1.61, m	1.61, m	24.3	24.3	1.60, m	1.60, m	24.8	24.8
4″	CH_2_	1.30, m	1.30, m	29.1	29.1	1.26, m	1.26, m	31.3	31.3
5″	CH_2_	1.29, m	1.29, m	29.1	29.1	1.30, m	1.30, m	21.9	21.9
6″	CH_2_	1.29, m	1.29, m	29.1	29.1				
6″	CH_3_					0.90, t (4.6)	0.90, t (4.6)	13.0	13.0
7″	CH_2_	1.29, m	1.29, m	29.1	29.1				
8″	CH_2_	1.29, m	1.29, m	29.1	29.1				
9″	CH_2_	1.29, m	1.29, m	29.1	29.1				
10″	CH_2_	1.28, m	1.28, m	31.6	31.3				
11″	CH_2_	1.30, m	1.30, m	22.1	22.1				
12″	CH_3_	0.90, t (4.6)	0.90, t (4.6)	12.8	12.8				
1‴	CO	-	-	167.2	167.2	-	-	167.0	167.0
2‴	C			128.3	128.3			128.3	128.3
3‴	CH	6.80, dd (6.8, 1.2)	6.80, dd (6.8, 1.2)	136.9	136.9	6.80, m	6.80, m	137.0	137.0
4‴	CH_3_	1.81, d (5.2)	1.81, d (5.2)	12.9	12.9	1.81, d (5.6)	1.81, d (5.6)	12.2	12.2
5‴	CH_3_	1.83, s	1.83, s	10.8	10.8	1.85	1.85		

^a^ Obtained from HSQC. Overlapping signal with deuterated methanol.

**Table 3:** ^1^H and ^13^C chemical shift values for compounds **5** and **6** in methanol-*d*_4_ (δ in ppm and *J* values in Hz in parentheses).

		5		6	
Position	Type	δ_H_ (*J*)	δ_C_	δ_H_ (*J*)	δ_C_
			(from HSQC and HMBC)		(from HSQC and HMBC)
1	CH	3.51, t (9.4)	72.7	3.51, dd (9.6, 4.6)	72.7
2	CH	5.36, t (10.0)	72.1	5.36, t (10.2)	72.1
3	CH	5.00, dd (10.4, 2.8)	70.1	5.03, dd (10.4, 2.8)	69.9
4	CH	5.50, brs	71.2	5.52, brs	71.2
5	CH	3.69, d (1.6)	69.5	3.68, d (5.6)	69.6
6	CH	3.68, brs	73.2	3.68, brs	73.2
1′	CO	-	167.3	-	167.3
2′	C	-	128.1	-	128.1
3′	CH	6.86, dd (7.2, 1.2)	137.6	6.88, dd (7.2, 1.2)	137.7
4′	CH_3_	1.79, d (7.2)	12.9	1.79, d (8.0)	12.9
5′	CH_3_	1.81, brs	10.7	1.81, brs	10.7
1″	CO	-	175.9	-	175.9
2″	CH	2.37, t (7.0)	33.8	2.20, t (7.0)	41.1
3″	CH_3_	1.04, d (6.8)	17.4		
3″	CH_2_			1.36, m	26.0
4″	CH_3_	1.00, d (7.2)	17.8	0.84, t (7.4)	10.5
5″	CH_3_			0.99, d (7.2)	15.6
1‴	CO	-	173.8	-	173.8
2‴	CH_2_	2.44, m	33.8	2.44, m	33.8
3‴	CH_2_	1.68, m	24.8	1.68, m	24.8
4‴	CH_2_	1.30, m	28.8	1.30, m	28.8
5‴	CH_2_	1.31, m	29.4	1.31, m	29.4
6‴	CH_2_	1.41, m	29.2	1.41, m	29.2
7‴	CH_2_	1.30, m	29.1	1.30, m	29.1
8‴	CH_2_	1.30, m	29.4	1.30, m	29.4
9‴	CH_2_	1.34, m	29.0	1.34, m	29.0
10‴	CH_2_	1.30, m	31.7	1.30, m	31.7
11‴	CH_2_	1.30, m	22.3	1.30, m	22.3
12‴	CH_3_	0.90, t (6.6)	13.0	0.90, t (6.4)	13.0

**Table 4:** Fluconazole reversal activity of isolated acylhexoses in *Saccharomyces cerevisiae* yeasts overexpressing the Mdr1 and Cdr1 transporters.

Compound	Minimum Effective Concentration (MEC, µM)	
	AD/CaMDR1	AD/CaCDR1
**1**	100	100
**2**	25	25
**3**	25	6.2
**4**	15	7.5
**5**	200	200
**6**	>200	>200
FK506	Nt	10

Nt: not tested.

**Table 5:** Effect of compounds **1**–**4** on Nile red intracellular accumulation in *Saccharomyces cerevisiae* strains overexpressing the Mdr1 and Cdr1 transporters.

Compound	Concentration (µM)	FIR		
		AD/CaMDR1	AD/CaCDR1	AD1-8u^−^
**1**	50	1.04 ± 0.00 **	0.97 ± 0.05	1.01 ± 0.01
	25	1.05 ± 0.04		
**2**	50	1.93 ± 0.43 *	3.78 ± 0.49 **	0.92 ± 0.02
	25	1.15 ± 0.16	1.56 ± 0.15	
**3**	50	3.86 ± 0.02 **	6.27 ± 1.47 **	1.00 ± 0.02
	25	0.94 ± 0.04	2.48 ± 0.45 *	
	12.5		1.69 ± 0.09 **	
	6.2		1.42 ± 0.11	
**4**	62	6.83 ± 0.10 *	5.41 ± 0.86 **	0.97 ± 0.06
	31	3.53 ± 0.28 *	3.57 ± 0.68 *	
	15.5	1.36 ± 0.18	2.16 ± 0.41 *	
	7.8		1.45 ± 0.6	
FK506	20	Nt	1.45 ± 0.03 *	1.02 ± 0.03
	10	Nt	1.10 ± 0.07	

Fluorescence intensity ratio (FIR) = mean fluorescence intensity (MFI) of Nile red with compound/MFI of Nile red alone. Nt: not tested. The minimum effective concentrations (MECs), defined as the lowest concentrations showing a statistically significant difference in MFI compared with the untreated control, are indicated in bold for each compound. Statistically significant differences compared to the negative control were determined using a one-tailed paired Student’s *t*-test (** *p* < 0.01; * *p* < 0.05).

**Table 6:** Effect of compounds **3** and **4** isolated from *Solanum atriplicifolium* on the intracellular accumulation of rhodamine 6G in *Saccharomyces cerevisiae* AD/CaCDR1 yeast.

Compound	Concentration (µM)	FIF	
		*Saccharomyces cerevisiae* AD/CaCDR1	*Saccharomyces cerevisiae* AD1-8u^−^
**3**	50	6.59 ± 0.44 ***	0.96 ± 0.01
	25	5.76 ± 0.17 ***	
	12.5	1.55 ± 0.06 **	
	6.2	1.20 ± 0.14 *	
	**3.1**	1.26 ± 0.09 *	
	1.6	1.18 ± 0.14	
**4**	62	14.36 ± 1.02 **	0.95 ± 0.01
	31	12.65 ± 1.12 **	
	15.5	8.99 ± 2.32 *	
	7.8	8.26 ± 2.80 *	
	**3.9**	3.70 ± 0.44 *	
	1.9	0.88 ± 0.12	
FK506	**20**	2.59 ± 0.65 *	1.01 ± 0.02
	10	1.17 ± 0.09	

Fluorescence intensity ratio (FIR) = mean fluorescence intensity (MFI) of rhodamine 6G with compound/MFI of rhodamine 6G alone. The minimum effective concentrations (MECs), defined as the lowest concentrations showing a statistically significant difference in MFI compared with the untreated control, are indicated in bold for each compound. Meaningful differences compared to the negative control were determined using a one-tailed paired Student’s *t*-test (*** *p* < 0.001; ** *p* <0.01; * *p* < 0.05).

**Table 7:** Relative populations (%) of the α- and β-anomers obtained from mutarotation equilibrium calculations in water. Gibbs free energies and Boltzmann populations were calculated at 300 K.

	Relative Populations (%)			
	1	2	3	4
Isomer α	20	59	49	8
Isomer β	80	41	51	92

**Table 8:** Summary of free energies of binding (ΔG°_b_) obtained from molecular dynamics trajectories (MM-PBSA).

		ΔG°_b_ (kcal/mol)
Subject compounds (anomeric form) ^a^		
	**1**(α)	−39 ± 3
	**1(β)**	**−28 ± 1**
	**2** **(α)**	**−37 ± 2**
	**2**(β)	−35 ± 2
	**3**(α)	−42 ± 2
	**3**(β)	−37 ± 2
	**4**(α)	−36 ± 2
	**4(β)**	**−36 ± 2**
Reference inhibitor	milbemycin oxime	−36 ± 2
Cdr1 substrates	fluconazole	−22 ± 1
	Nile red	−23 ± 1
	rhodamine 6G	−32 ± 2

^a^ Values in bold whenever an anomeric form is largely most abundant.

## Data Availability

The original contributions presented in this study are included in the article/Supplementary Materials. Further inquiries can be directed to the corresponding authors.

## Supplementary Materials

The following supporting information can be downloaded at: https://www.mdpi.com/article/10.3390/plants15162523/s1, Figure S1. Liquid chromatography–mass spectrometry chromatogram of compound **5**. Figure S2. Liquid chromatography–mass spectrometry chromatogram of compound **6**. Figure S3. Liquid chromatography–mass spectrometry chromatogram of compound **1**. Figure S4. Liquid chromatography–mass spectrometry chromatogram of compound **2**. Figure S5. Liquid chromatography–mass spectrometry chromatogram of compound **3**. Figure S6. Liquid chromatography–mass spectrometry chromatogram of compound **4**. Figure S7. Flow cytometric analysis of the effect of compound **1** at 50 μM on Nile red accumulation in Saccharomyces cerevisiae AD/CaMDR1. The representative histogram shows the intracellular fluorescence intensity of cells treated with Nile red alone (blue) and cells treated with Nile red in combination with compound **1** (red). Figure S8. Flow cytometric analysis of the effect of compound **2** at 50 μM on Nile red accumulation in (A) Saccharomyces cerevisiae AD/CaMDR1 and in (B) S. cerevisiae AD/CaCDR1. The representative histograms show the fluorescence intensity of cells treated with Nile red alone (blue) and cells treated with Nile red in combination with compound **2** (red). Figure S9. Flow cytometric analysis of the effect of compound **3** on Nile red accumulation in (A) Saccharomyces cerevisiae AD/CaMDR1 treated with 50 μM and in (B) S. cerevisiae AD/CaCDR1 treated with compound **3** at concentrations ranging from 50 to 12.5 μM. The representative histograms show the intracellular fluorescence intensity of cells treated with Nile red alone (blue) and cells treated with Nile red in combination with compound **3** (red). Figure S10. Flow cytometric analysis of the effect of compound **4** on intracellular Nile red accumulation in (A) Saccharomyces cerevisiae AD/CaMDR1 treated with compound **4** at 62 and 31 μM and in (B) S. cerevisiae AD/CaCDR1 treated with compound **4** at 62 and 15.5 μM. The representative histograms show the fluorescence intensity of cells treated with Nile red (blue) and cells treated with Nile red and compound **4** (red). Figure S11. Flow cytometric analysis of the effect of (A) compound **3** at 50 and 3.1 μM and (B) compound **4** at 62 and 3.9 μM on intracellular rhodamine 6G accumulation in Saccharomyces cerevisiae AD/CaCDR1 cells. Representative histograms show the fluorescence intensity of cells treated with rhodamine 6G alone (blue) and cells treated with rhodamine 6G in combination with compounds **3** or **4** (red). Figure S12. A typical RMSD for a 120 ns trajectory obtained for compound **4**β. All heavy atoms computed for the ligand and surrounding amino acids within 5Å. Figure S13. Superimposition of representative structures from the most populated cluster of the reference crystallographic inhibitor milbemycin oxime (yellow), compound **3**β (green), compound **2**α (brown) and compound **1**β (gray). The poorer overlap between the reference inhibitor and the less active compound **1**β is noted. Figure S14. Structure at the center of the most populated cluster from the 100 ns cluster analysis for 3β (light blue). The translucent yellow surface shows the region occupied by the reference inhibitor milbemycin from a similar cluster analysis. Note that almost the entire region is occupied by the studied compound. Figure S15. Superimposition of representative structures from the most populated cluster of compound **3**β (green), with Nile red (red) and rhodamine 6G (orange). The subject compound showed a clear overlap with the binding poses of both dyes, with a slightly better agreement observed for rhodamine 6G. Figure S16. Analysis of the contribution to the ΔG°b in terms of per-residue decomposition. Profiles for the most powerful ligands (shown in main text Figure 11A) and one of the poorest, compound **1**β. Note the difference in most relevant peaks. Figure S17. Analysis of the contribution to the ΔG°b in terms of per-residue decomposition. Profiles for the dyes Nile red and rhodamine 6G. Figure S18. Comparison of the most active compounds 3 and 4β (above, also shown in main text) with the less active compound **1**β (below). Note the lack or shortened peaks for compound **1**β. Figure S19. Left panel: two views of the complex Cdr1/fluconazole embedded in membrane (as in main text Figure 12. Right panel: superimposition of the three most populated clusters from the simulations with the custom setup (color rainbow from red to yellow) with the three most populated clusters of the full membrane-embedded simulation (rainbow from green to violet). Figure S20.Snapshot of the most populated cluster of the Cdr1/fluconazole complex in membrane (red), including lipids polar heads (hydrophobic chains, ergosterol molecules, water and ions not shown). Superimposed the most populated cluster of Cdr1/3α complex in membrane (green), only backbone (ribbons) and ligand (licorice). The inset shows a zoomed view of a −90° rotation of the z axis. Figure S21. RMSd of the equilibration trajectory of the complex Cdr1/fluconazole embedded in the cell membrane. Blue: all-atoms of the protein and ligand. Red: all heavy atoms of fluconazole and surrounding residues within 5 Å. Figure S22. Analysis of the decomposition per residue of the contributions to the ΔG°b as in main text Figure 11. Comparison of the patterns obtained from the simpler constrained (magenta) and explicit realistic membrane (cyan) simulations for fluconazole (A) and compound **3**α (B). Figure S23. Bioguided-isolation scheme of compounds obtained from Solanum atriplicifolium. Table S1. Summary of DFT calculations on the subject compounds in their different anomeric forms.
