# Supplementary material for: New Acylglucoses from Solanum atriplicifolium as Multidrug Resistance Reversal Agents Targeting Cdr1 and Mdr1
Source: Plants (Basel). 2026 Aug 20;15(16):2523. doi: 10.3390/plants15162523 (PMC13516204; doi:10.3390/plants15162523)
Supplement: Supplementary file 1 [file plants-15-02523-s001.zip › plants-4471021-supplementary.pdf]

Supplementary material

New acylglucoses from *Solanum atriplicifolium* as multidrug resistance reversal agents targeting Cdr1 and Mdr1

Florimar Gil [1], Erika Ripani [2], Mariana Belén Joray [1], María Paula del Valle [2], Richard D. Cannon [3], Constantinos Athanassopoulos [4], D. Mariano A. Vera [2,5] and María Cecilia Carpinella [1*]**

[1] Fine Chemical and Natural Products Laboratory, CIDIE CONICET-UCC, Universidad Católica de Córdoba, Córdoba, Argentina
[2] Department of Chemistry and Biochemistry, College of Exact and Natural Sciences, National University of Mar del Plata - QUIAMM – INBIOTEC CONICET, Mar del Plata, Argentina.
[3] Sir John Walsh Research Institute, University of Otago, School of Dentistry, PO Box 647, Dunedin 9054, New Zealand.
[4] Laboratory of Synthetic Organic Chemistry, Department of Chemistry, University of Patras, GR-26504, Patras, Greece.
[5] Institute of Chemistry and Biochemistry of Mar del Plata (IQUIBIM) – Universidad Nacional de Mar del Plata.
[*] Correspondence: ceciliacarpinella@ucc.edu.ar (M.C.C.); dmavera@yahoo.com (D.M.A.V.)

Liquid chromatography–mass spectrometry chromatograms of atriplicifolin A (1), atriplicifolin B (2), atriplicifolin C (3), atriplicifolin D (4), atriplicifolin E (5) and atriplicifolin F (6):

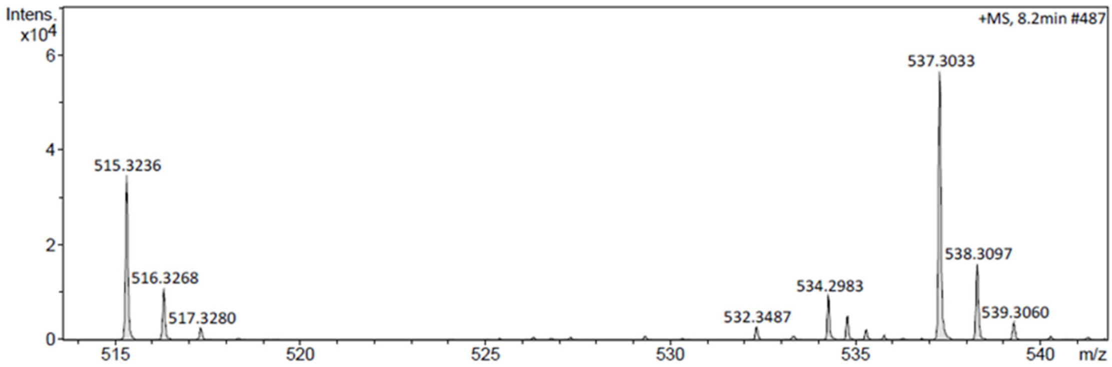

Meas. m/z	#	Ion Formula	m/z	err [ppm]	mSigma	# mSigma	Score	rdb	e⁻ Conf	N-Rule
515.3236	1	C27H47O9	515.3215	-4.2	7.4	1	100.00	4.5	even	ok
537.3033	1	C27H46NaO9	537.3034	0.2	9.5	1	100.00	4.5	even	ok

Figure S1. Liquid chromatography–mass spectrometry chromatogram of compound **5.**

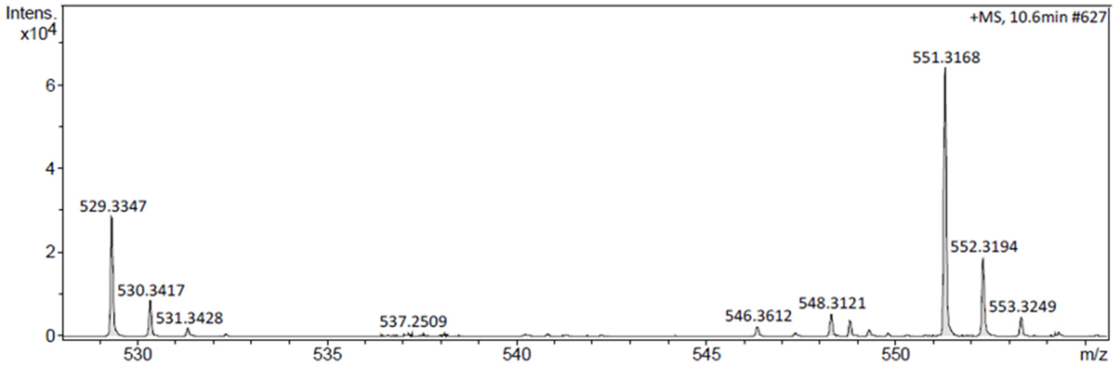

Meas. m/z	#	Ion Formula	m/z	err [ppm]	mSigma	# mSigma	Score	rdb	e⁻ Conf	N-Rule
529.3347	1	C28H49O9	529.3371	4.6	7.2	1	100.00	4.5	even	ok
551.3168	1	C28H48NaO9	551.3191	4.1	10.6	1	100.00	4.5	even	ok

Figure S2. Liquid chromatography–mass spectrometry chromatogram of compound **6.**

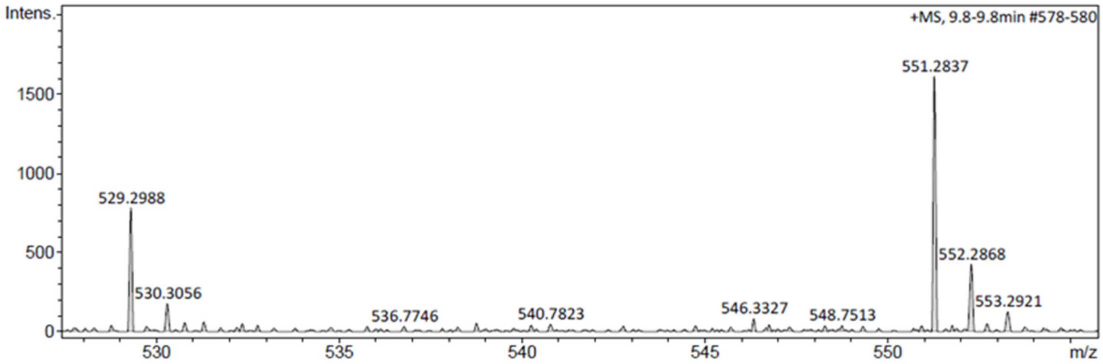

Meas. m/z	#	Ion Formula	m/z	err [ppm]	mSigma	# mSigma	Score	rdb	e¯ Conf	N-Rule
529.2988	1	C27H45O10	529.3007	3.6	38.1	2	34.47	5.5	even	ok
551.2837	1	C27H44NaO10	551.2827	-1.9	20.6	1	100.00	5.5	even	ok

Figure S3. Liquid chromatography–mass spectrometry chromatogram of compound **1**.

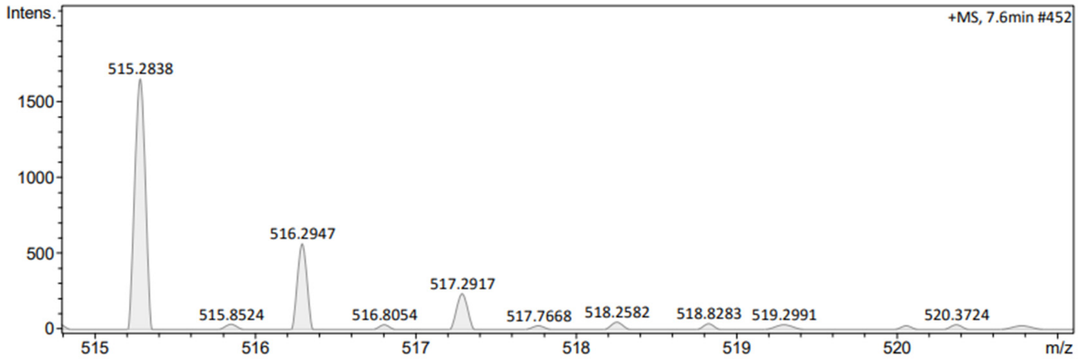

Meas. m/z	#	Ion Formula	m/z	err [ppm]	mSigma	# mSigma	Score	rdb	e¯ Conf	N-Rule
515.2838	1	C26H43O10	515.2851	2.5	48.8	1	100.00	5.5	even	ok

Figure S4. Liquid chromatography–mass spectrometry chromatogram of compound **2.**

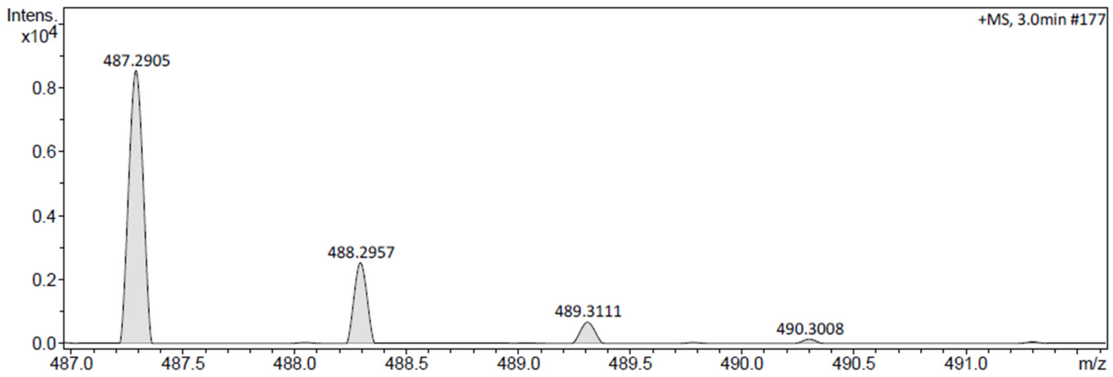

Meas. m/z	#	Ion Formula	m/z	err [ppm]	mSigma	# mSigma	Score	rdb	e¯ Conf	N-Rule
487.2905	1	C25H43O9	487.2902	-0.8	29.5	1	100.00	4.5	even	ok

Figure S5. Liquid chromatography–mass spectrometry chromatogram of compound **3.**

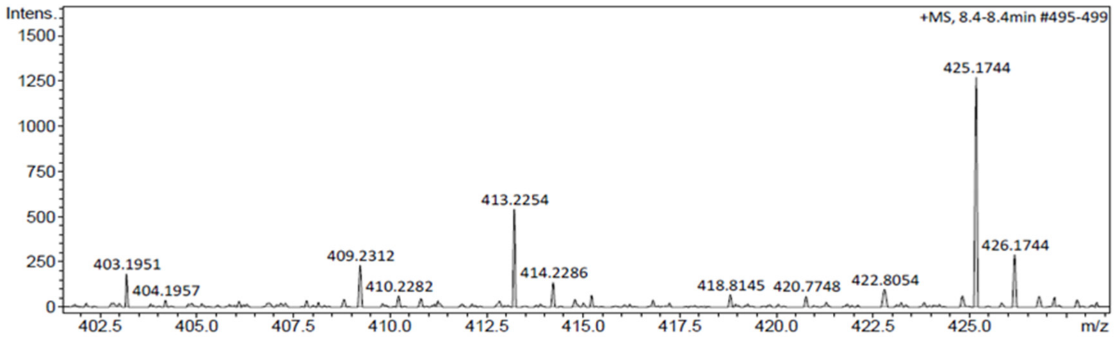

Meas. m/z	#	Ion Formula	m/z	err [ppm]	mSigma	# mSigma	Score	rdb	e¯ Conf	N-Rule
403.1951	1	C19H31O9	403.1963	2.8	23.9	1	100.00	4.5	even	ok
425.1744	1	C19H30NaO9	425.1782	8.9	25.0	1	9.08	4.5	even	ok

Figure S6. Liquid chromatography–mass spectrometry chromatogram of compound **4.**

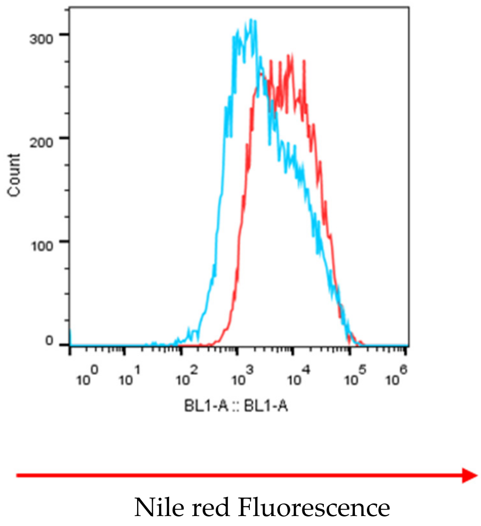

1

Figure S7. Flow cytometric analysis of the effect of compound **1** at 50 µM on Nile red accumulation in *Saccharomyces cerevisiae* AD/CaMDR1. The representative histogram shows the intracellular fluorescence intensity of cells treated with Nile red alone (blue) and cells treated with Nile red in combination with compound **1** (red).

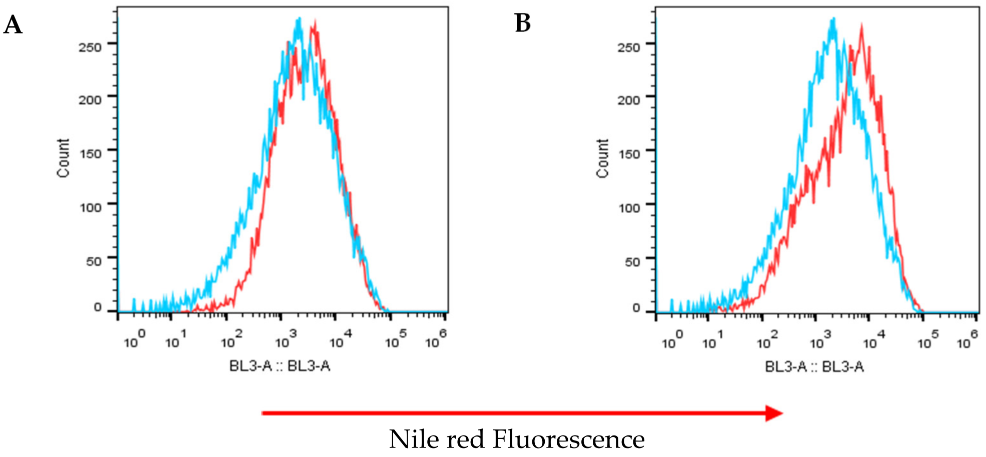

2

Figure S8. Flow cytometric analysis of the effect of compound **2** at 50 µM on Nile red accumulation in (A) *Saccharomyces cerevisiae* AD/CaMDR1 and in (B) *S. cerevisiae* AD/CaCDR1. The representative histograms show the fluorescence intensity of cells treated with Nile red alone (blue) and cells treated with Nile red in combination with compound **2** (red).

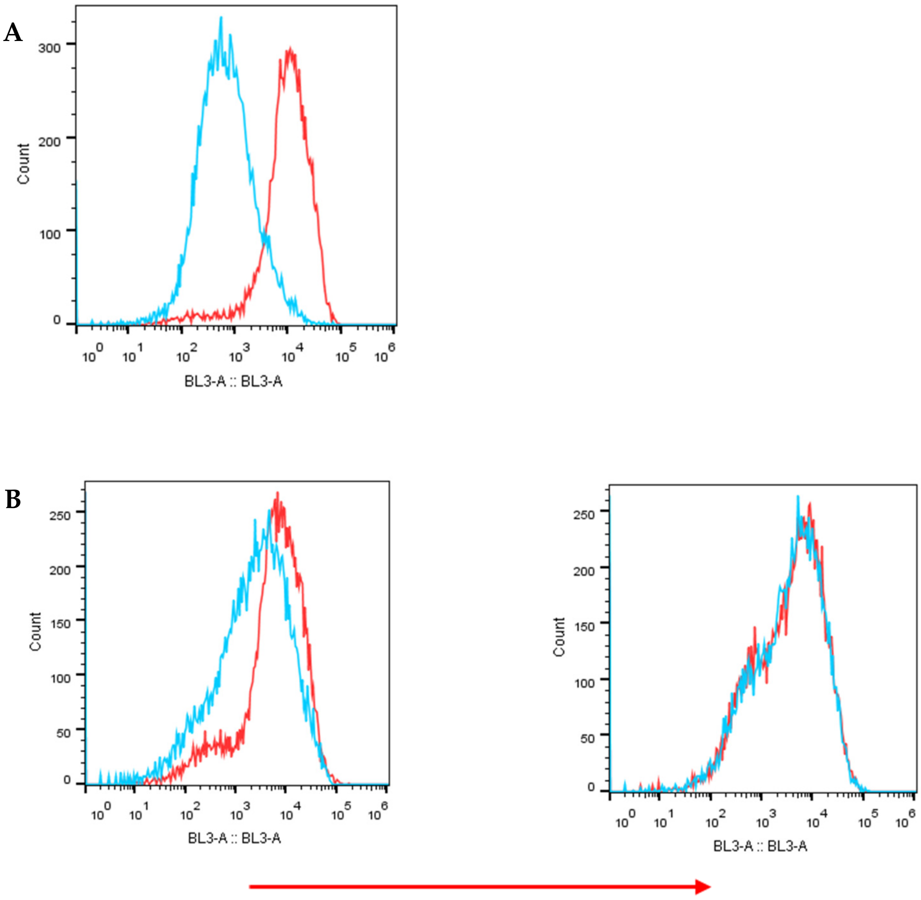

Nile red Fluorescence

3

Figure S9. Flow cytometric analysis of the effect of compound **3** on Nile red accumulation in (A) *Saccharomyces cerevisiae* AD/CaMDR1 treated with 50 µM and in (B) *S. cerevisiae* AD/CaCDR1 treated with compound **3** at concentrations ranging from 50 to 12.5 µM. The representative histograms show the intracellular fluorescence intensity of cells treated with Nile red alone (blue) and cells treated with Nile red in combination with compound **3** (red).

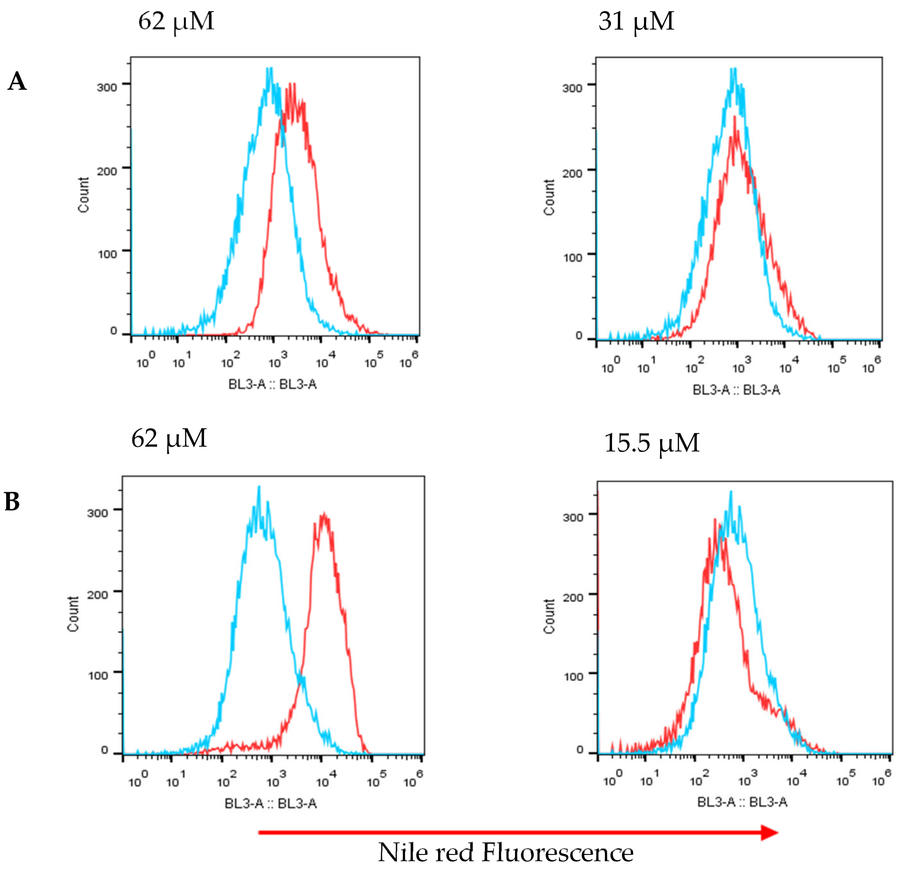

Figure S10. Flow cytometric analysis of the effect of compound **4** on intracellular Nile red accumulation in (A) *Saccharomyces cerevisiae* AD/CaMDR1 treated with compound **4** at 62 and 31 µM and in (B) *S. cerevisiae* AD/CaCDR1 treated with compound **4** at 62 and 15.5 µM. The representative histograms show the fluorescence intensity of cells treated with Nile red (blue) and cells treated with Nile red and compound **4** (red).

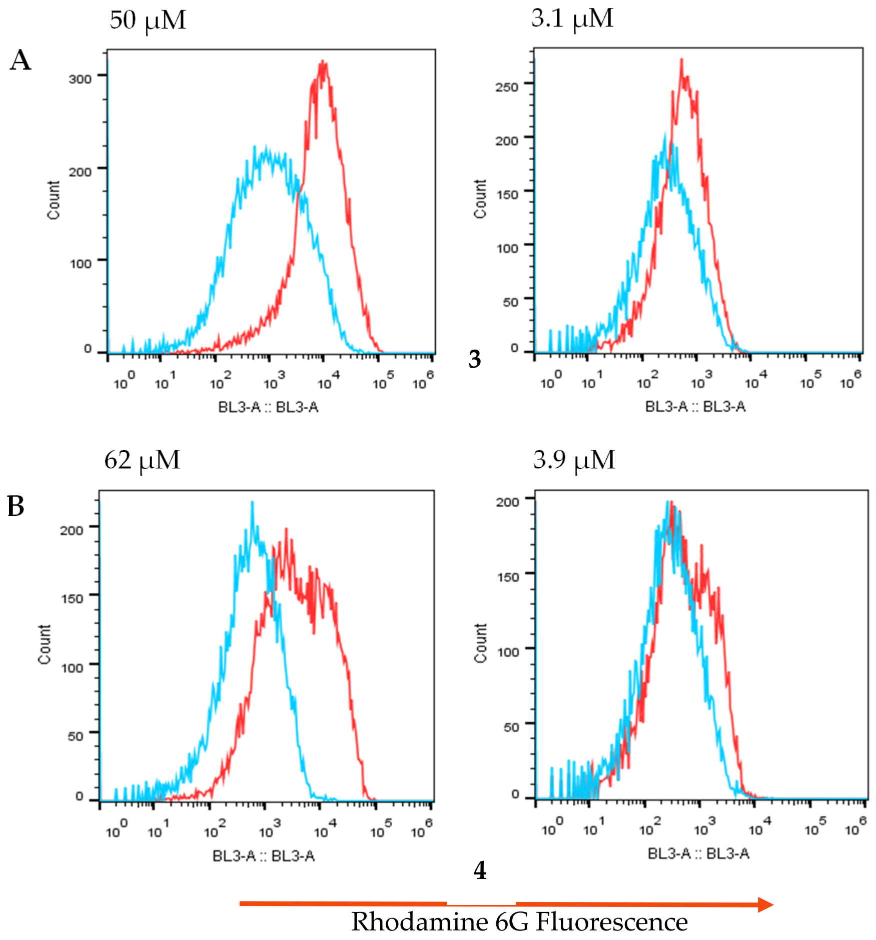

Figure S11. Flow cytometric analysis of the effect of (A) compound **3** at 50 and 3.1 μM and (B) compound **4** at 62 and 3.9 μM on intracellular rhodamine 6G accumulation in *Saccharomyces cerevisiae* AD/CaCDR1 cells. Representative histograms show the fluorescence intensity of cells treated with rhodamine 6G alone (blue) and cells treated with rhodamine 6G in combination with compounds **3** or **4** (red).

Computational Details

Details regarding the quantum chemistry calculations for the determination of the most abundant anomers of the subject compounds

Calculations were done at the CAM-B3LYP/6-31G(d,p) level of theory using SMD implicit solvation model for water at 298.15 K and 1 atm pressure. Thermochemistry from the Hessian matrix diagonalization and harmonic frequencies analyses. The Gibbs free energy differences, ΔG°(β-α), were used to calculate the Boltzman populations reported in the main text (Table 7).

Table S1. Summary of DFT calculations on the subject compounds in their different anomeric forms.

	Electronic Energy /Hartree	Electronic Energy + Z.P.E. /Hartree	Total Free Energy /Hartree	ΔG°=G°(β)-G°(α) / (kcal/mol)
**1**α	-1806.896841	-1806.194244	-1806.276020	
**1**β	-1806.895993	-1806.194243	-1806.277348	-0.833
**2**α	-1767.602744	-1766.928733	-1767.008368	
**2**β	-1767.601758	-1766.928271	-1767.008026	0.215
**3**α	-1654.309022	-1653.645675	-1653.726565	
**3**β	-1654.307966	-1653.645237	-1653.726592	-0.017
**4**α	-1418.562805	-1418.070861	-1418.138680	
**4**β	-1418.564230	-1418.073316	-1418.141023	-1.470

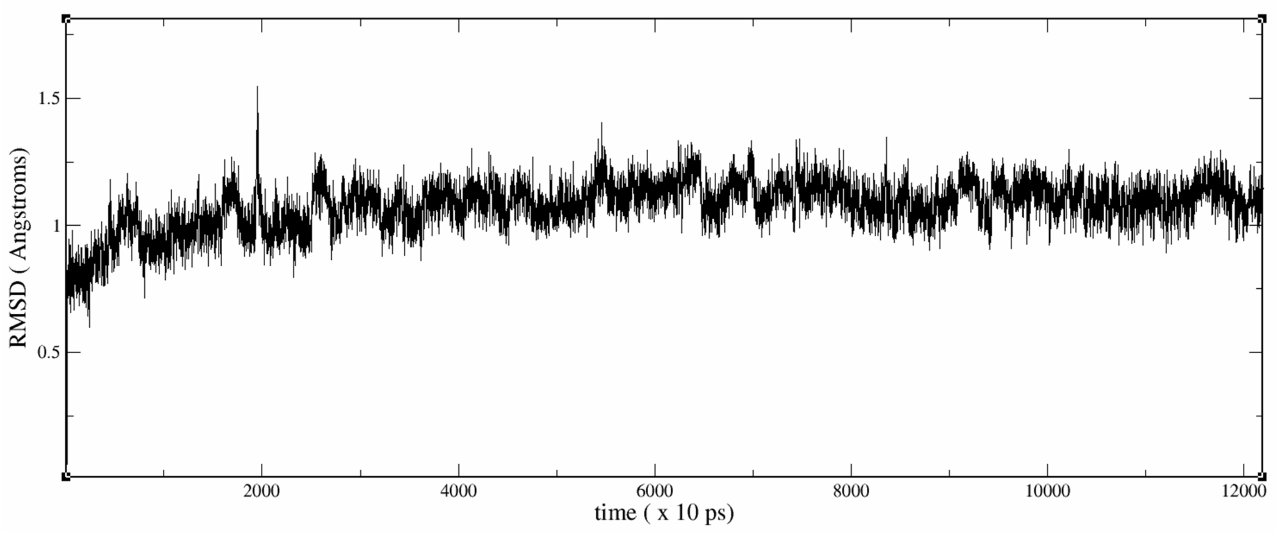

Figure S12. A typical RMSD for a 120 ns trajectory obtained for compound **4**β. All heavy atoms computed for the ligand and surrounding amino acids within 5Å.

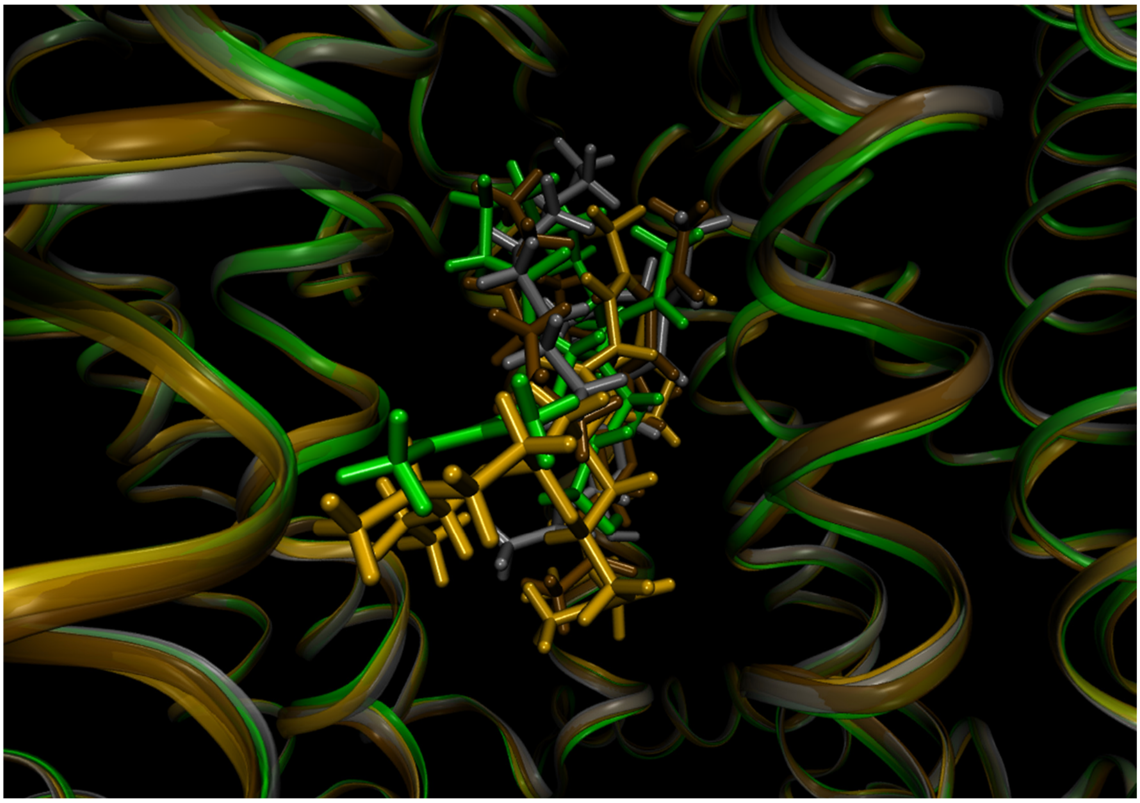

Figure S13. Superimposition of representative structures from the most populated cluster of the reference crystallographic inhibitor milbemycin oxime (yellow), compound **3**β (green), compound **2**α (brown) and compound **1**β (gray). The poorer overlap between the reference inhibitor and the less active compound **1**β is noted.

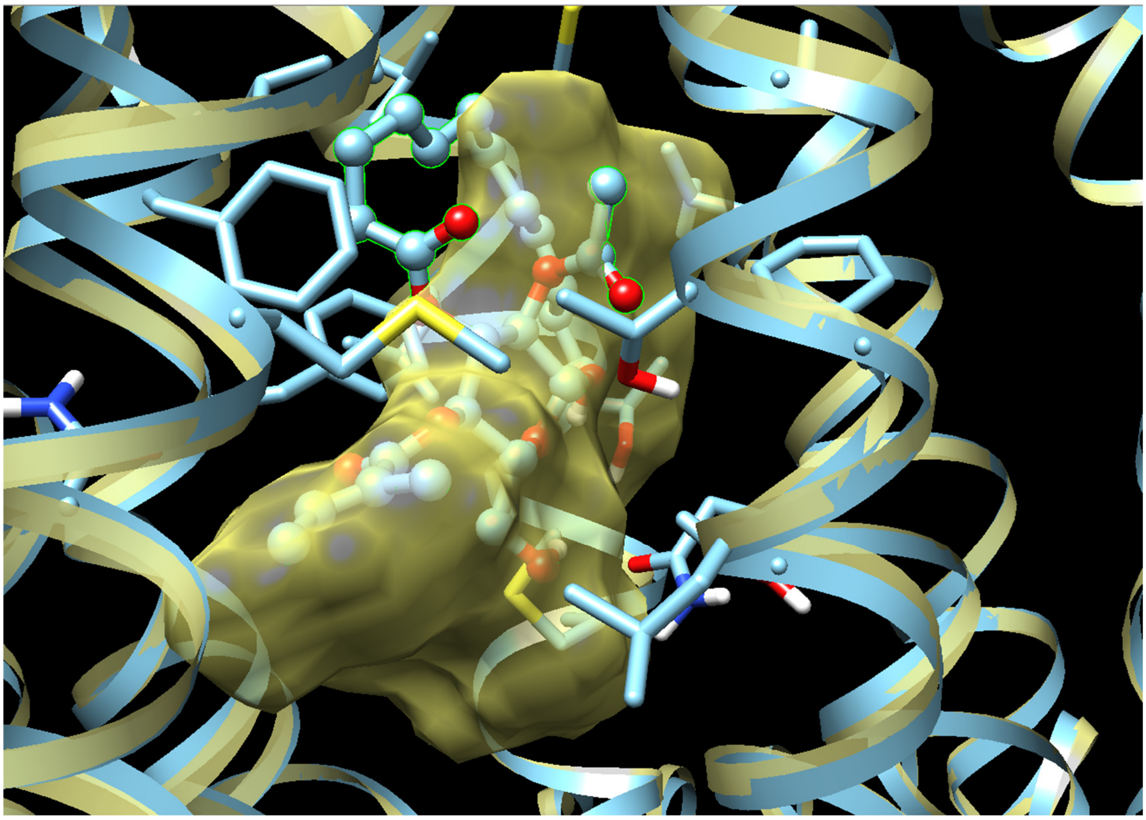

Figure S14. Structure at the center of the most populated cluster from the 100 ns cluster analysis for **3β** (light blue). The translucent yellow surface shows the region occupied by the reference inhibitor milbemycin from a similar cluster analysis. Note that almost the entire region is occupied by the studied compound.

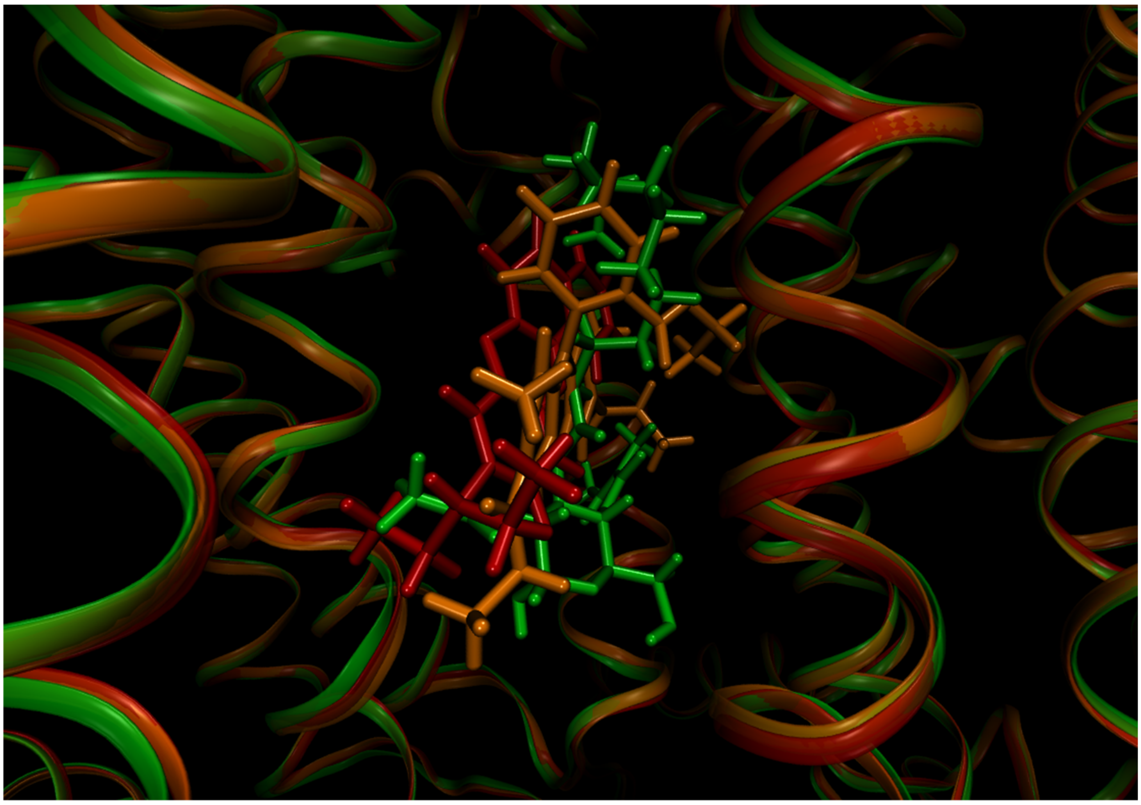

Figure S15 Superimposition of representative structures from the most populated cluster of compound **3β** (green), with Nile red (red) and rhodamine 6G (orange). The subject compound showed a clear overlap with the binding poses of both dyes, with a slightly better agreement observed for rhodamine 6G.

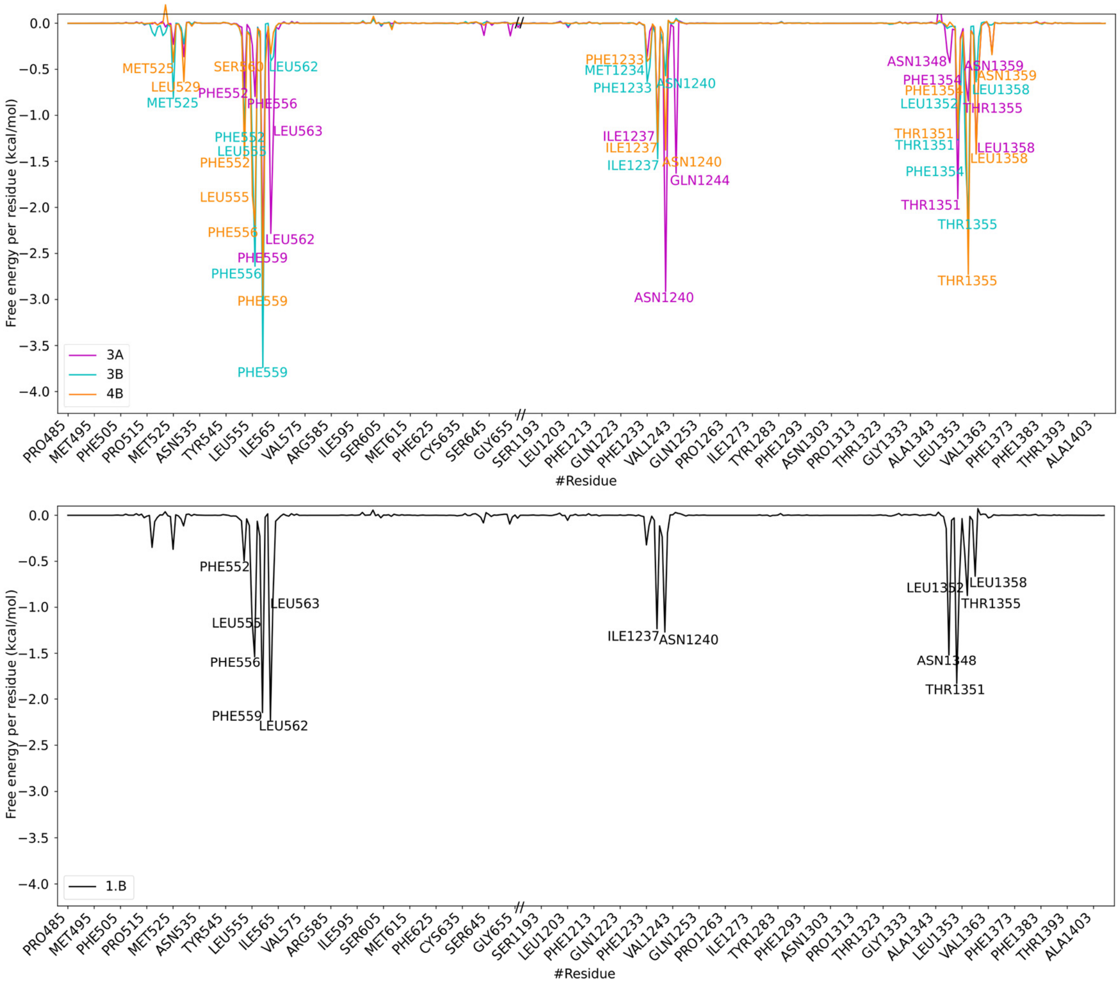

Figure S16. Analysis of the contribution to the $\Delta G°_b$ in terms of per-residue decomposition. Profiles for the most powerful ligands (shown in main text Figure 11A) and one of the poorest, compound 1β. Note the difference in most relevant peaks.

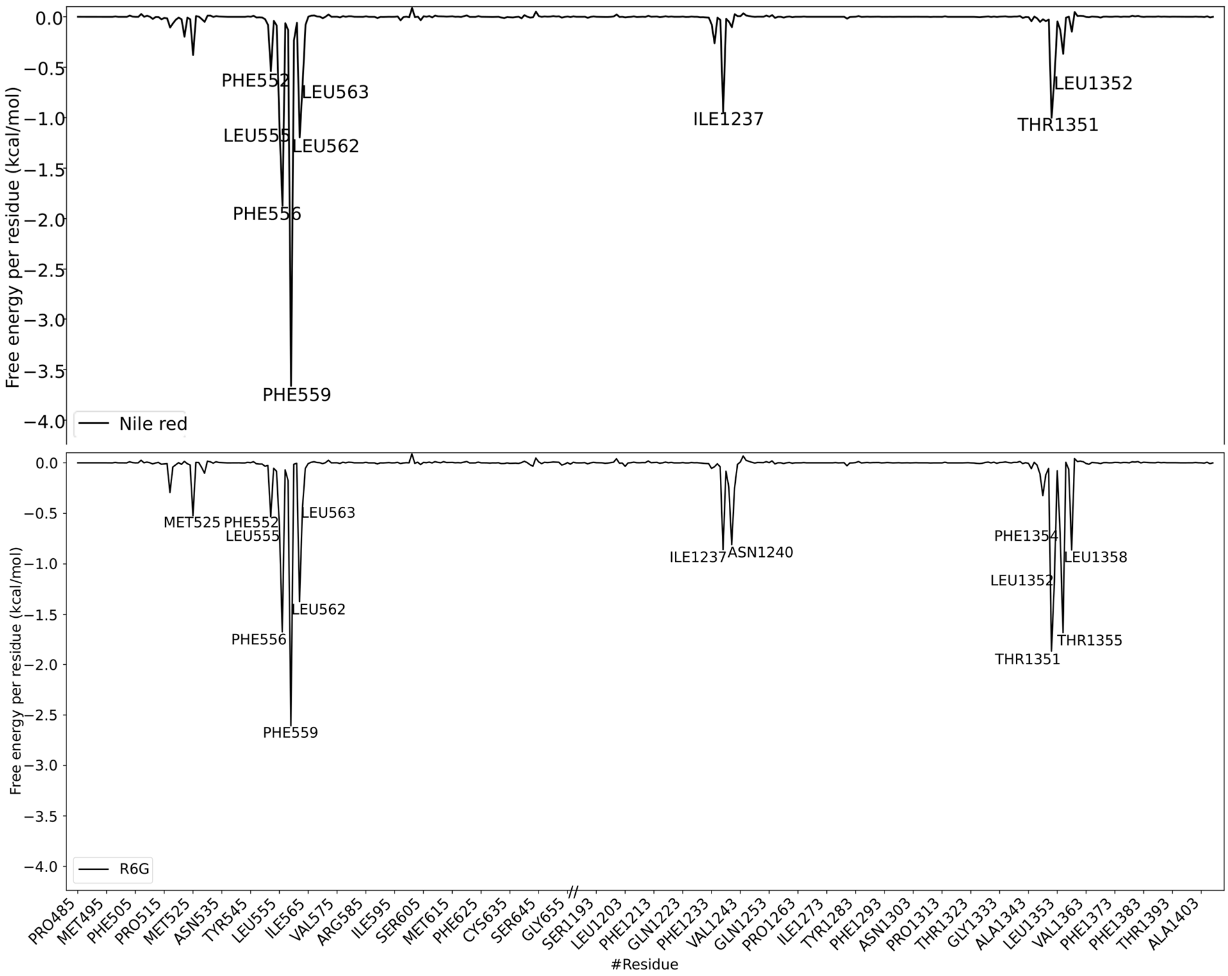

Figure S17. Analysis of the contribution to the $\Delta G°_b$ in terms of per-residue decomposition. Profiles for the dyes Nile red and rhodamine 6G.

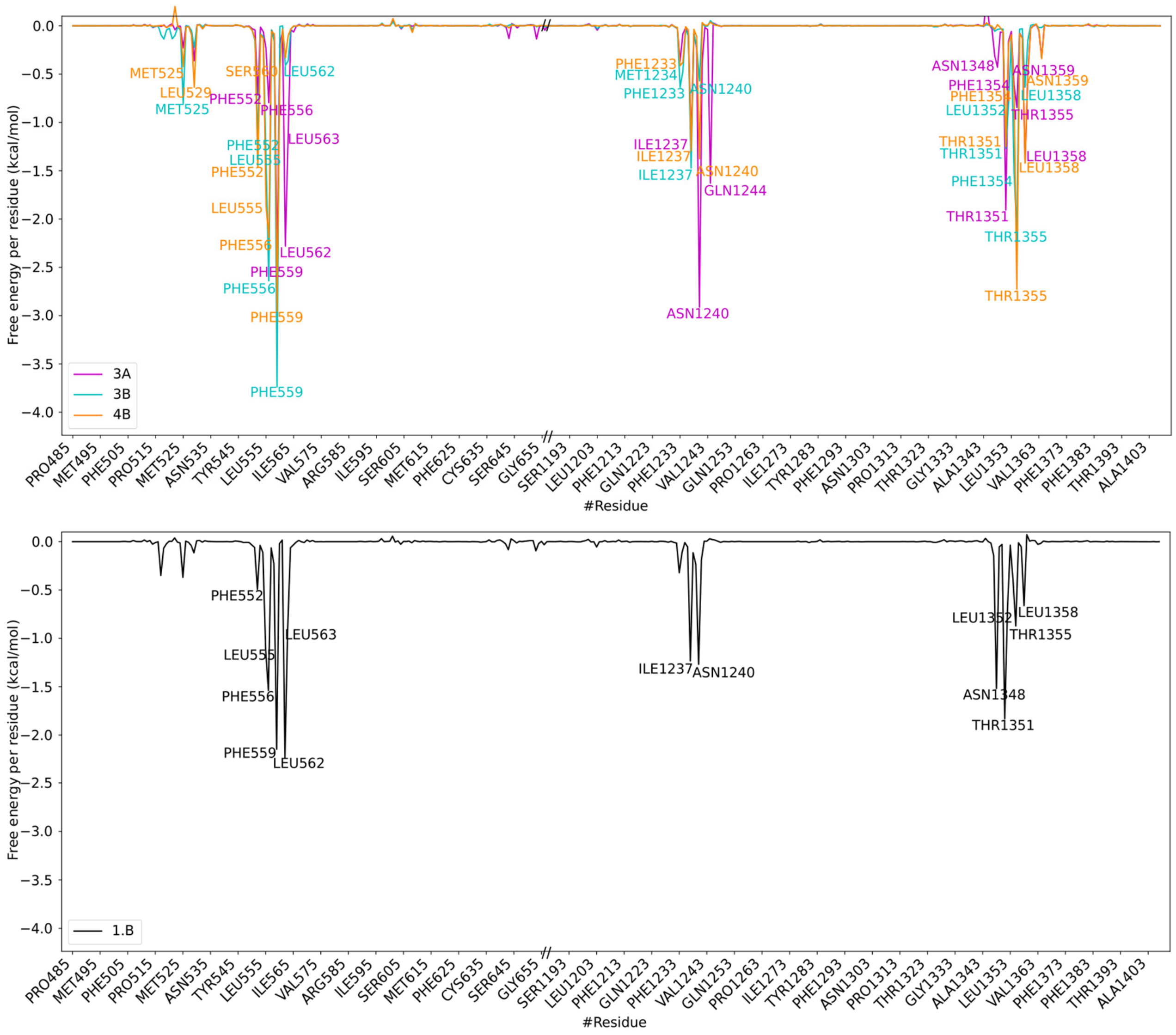

Figure S18. Comparison of the most active compounds **3** and **4**β (above, also shown in main text) with the less active compound **1**β (below). Note the lack or shortened peaks for compound **1**β.

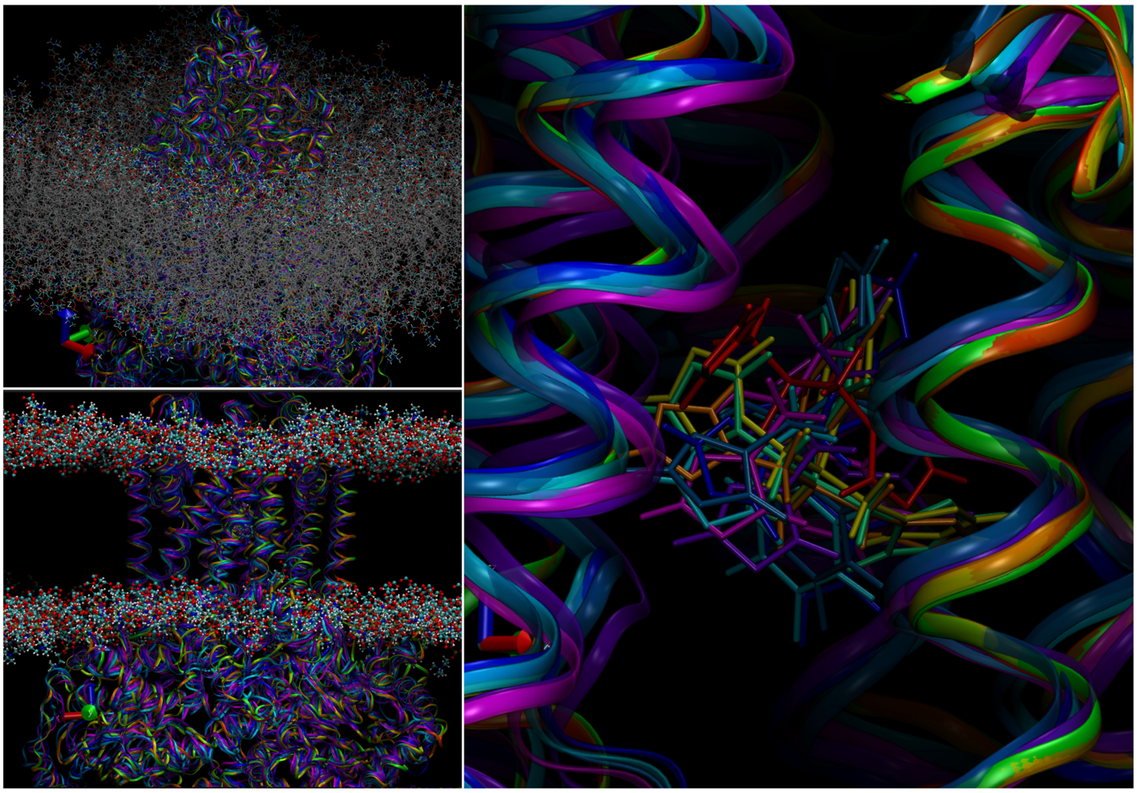

Figure S19. Left panel: two views of the complex Cdr1/fluconazole embedded in membrane (as in main text Figure 12. Right panel: superimposition of the three most populated clusters from the simulations with the custom setup (color rainbow from red to yellow) with the three most populated clusters of the full membrane-embedded simulation (rainbow from green to violet)

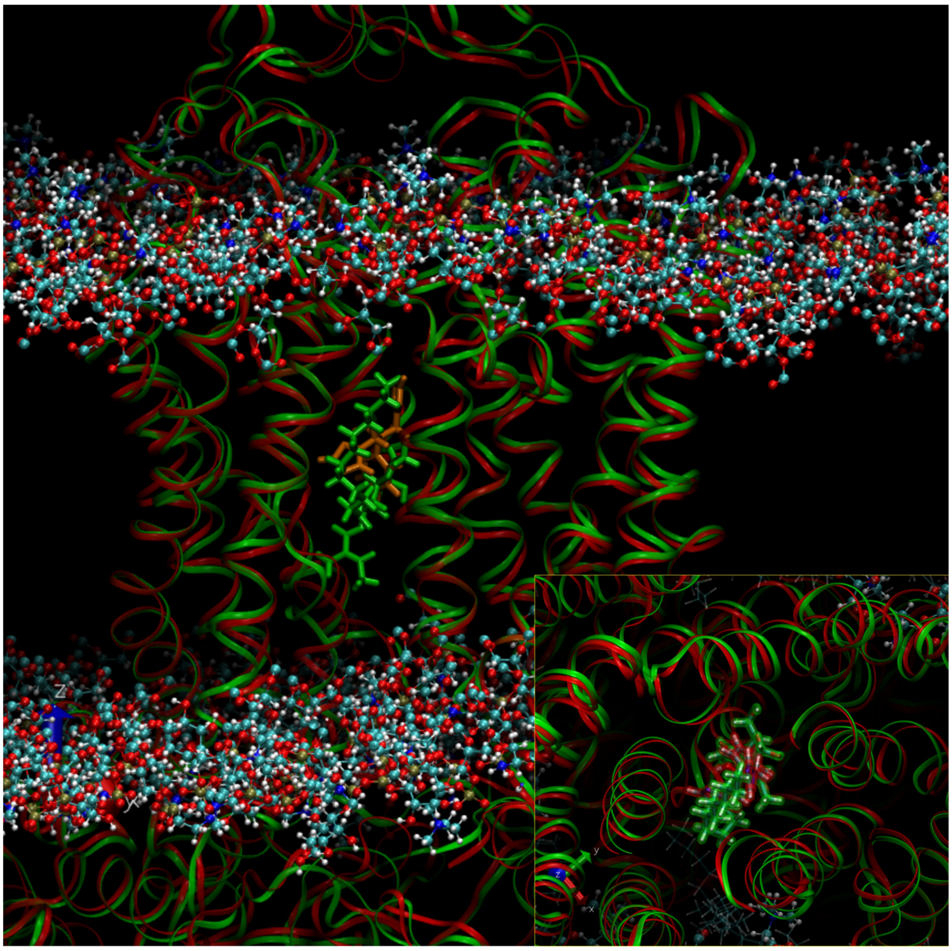

Figure S20.Snapshot of the most populated cluster of the Cdr1/fluconazole complex in membrane (red), including lipids polar heads (hydrophobic chains, ergosterol molecules, water and ions not shown). Superimposed the most populated cluster of Cdr1/**3α** complex in membrane (green), only backbone (ribbons) and ligand (licorice). The inset shows a zoomed view of a -90° rotation of the *z* axis.

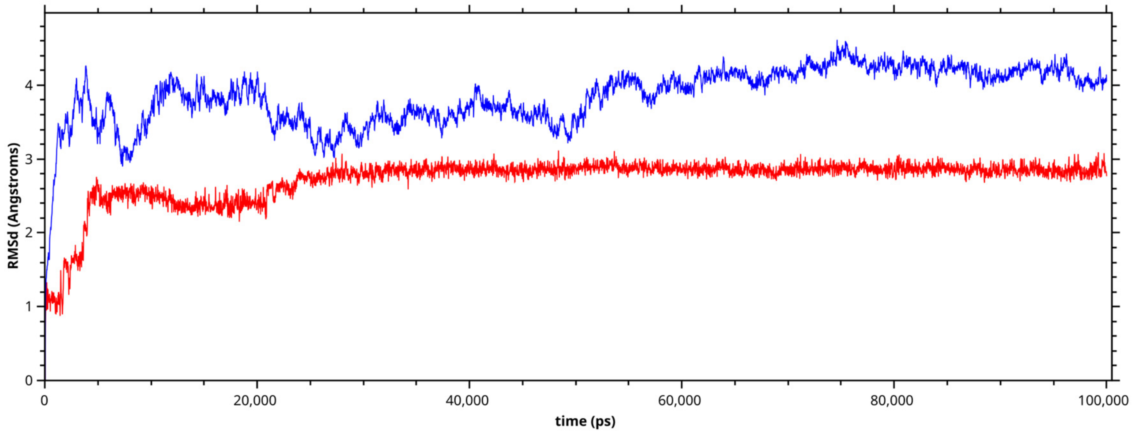

Figure S21. RMSd of the equilibration trajectory of the complex Cdr1/fluconazole embedded in the cell membrane. Blue: all-atoms of the protein and ligand. Red: all heavy atoms of fluconazole and surrounding residues within 5 Å.

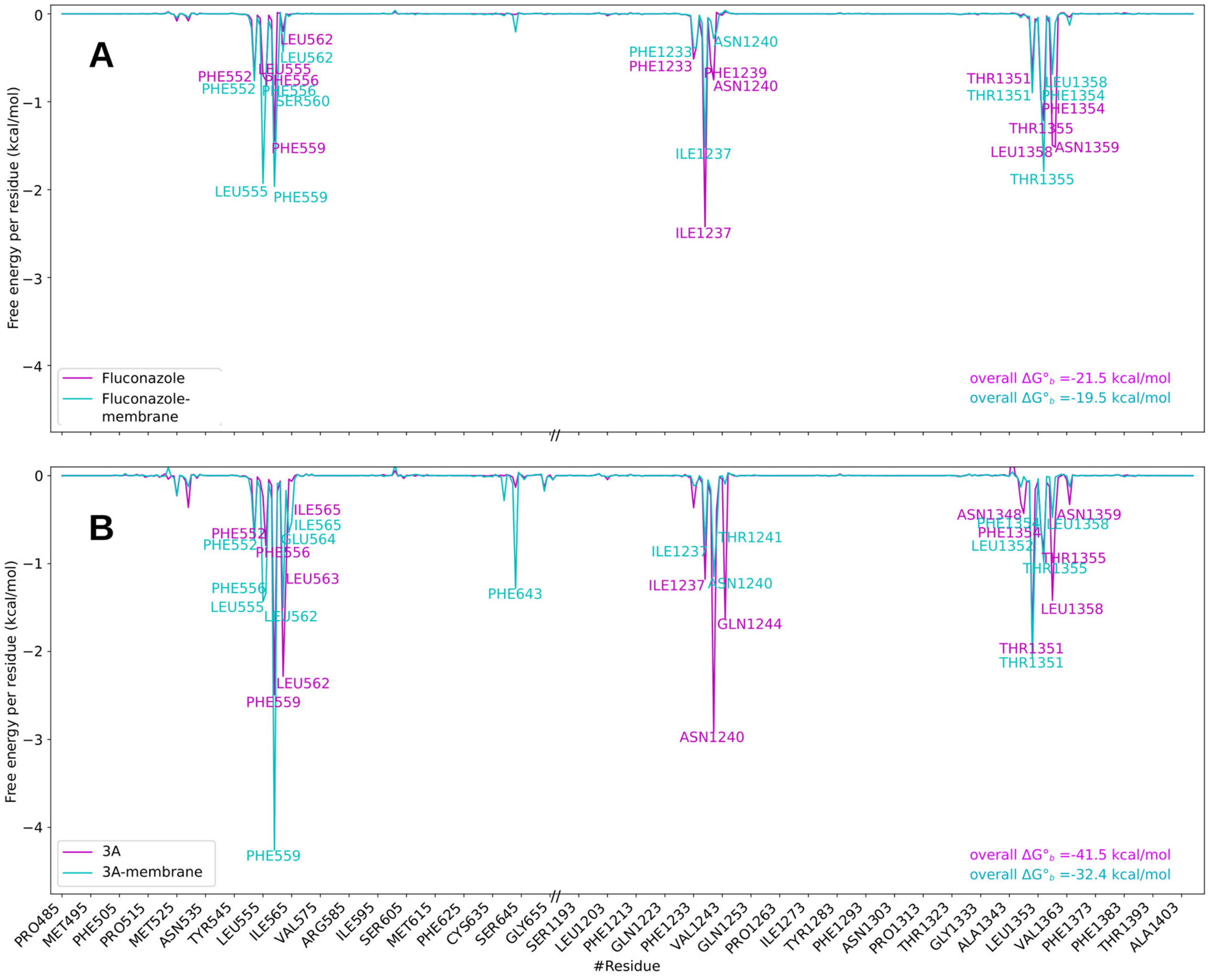

Figure S22. Analysis of the decomposition per residue of the contributions to the ΔG°$_b$ as in main text Figure 11. Comparison of the patterns obtained from the simpler constrained (magenta) and explicit realistic membrane (cyan) simulations for fluconazole (**A**) and compound **3*α*** (**B**).

Bioguided-isolation scheme

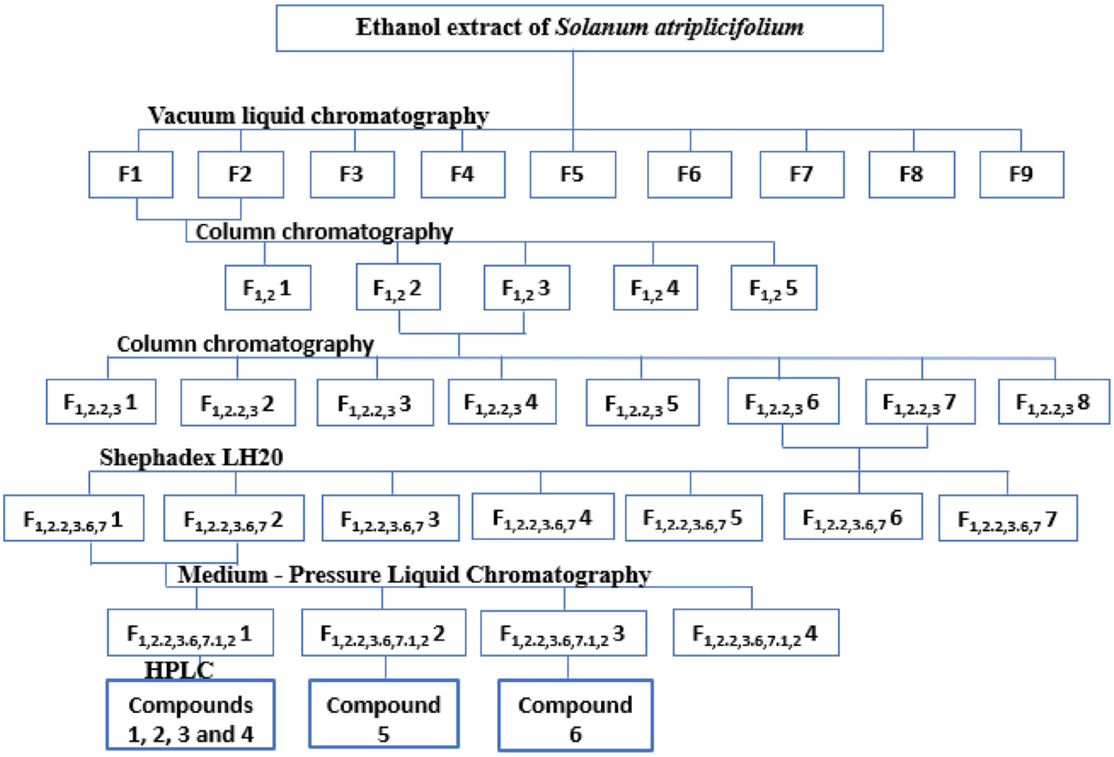

Figure S23. Bioguided-isolation scheme of compounds obtained from *Solanum atriplicifolium*.
